# Non-Targeted and Targeted Screening of Organic Contaminants in Honeybees’ Death Incidents in Greece: A Story Beyond Pesticides

**DOI:** 10.3390/jox16020064

**Published:** 2026-04-08

**Authors:** Eirini Baira, Evangelia N. Tzanetou, Electra Manea-Karga, Kyriaki Machera, Konstantinos M. Kasiotis

**Affiliations:** Laboratory of Pesticides’ Toxicology, Scientific Directorate of Pesticides Control and Phytopharmacy, Benaki Phytopathological Institute, 14561 Kifissia, Greece; ev.tzanetou@bpi.gr (E.N.T.); e.manea-karga@bpi.gr (E.M.-K.); k.machera@bpi.gr (K.M.)

**Keywords:** honeybees, chemical contamination, HRMS, risk assessment, pesticide residue analysis

## Abstract

Despite the undisputable ecosystem importance of honeybees, human activities have a substantial impact on their health. Since foraging is directly linked to a wide range of crops and bee-attracting flowers, plant protection products are at the forefront of chemical scrutiny, along with contamination of pollen, nectar, beehive components and water by other xenobiotics. In this study, a non-targeted Liquid Chromatography-High-Resolution Mass Spectrometry (LC-HRMS) screening was applied to 25 honeybee samples collected after reported death incidents in Greece. This approach led to the tentative annotation of over 50 compounds across various chemical classes, including pesticides, PFAS candidates not included in the EFSA “PFAS-4”, pharmaceuticals, antibiotics, industrial chemicals, and natural product constituents. In parallel, targeted pesticide residue analysis using liquid and gas chromatography coupled to tandem mass spectrometry (LC-MS/MS and GC-MS/MS) was performed, covering more than 250 active substances and providing direct quantitative results, revealing 11 active substances in concentrations ranging from <limit of quantification (LOQ) to 0.95 mg/kg, overlapping substantially with the HRMS detection. Overall, this study does not allow concrete causal attribution of mortality to specific chemicals; however, it documents complex co-occurrence patterns (pesticides together with other xenobiotics and plant bioactives), not excluding sublethal and mixture-toxicity effects. Quantified pesticide concentrations were below acute LD50-based thresholds, yet selected samples combined neonicotinoid/pyrethroid/fungicide signatures and other contaminants, supporting the need for mixture-toxicity frameworks and effect-based follow-ups.

## 1. Introduction

Honeybees are indispensable contributors to both natural ecosystems and agricultural production, particularly as pollinators of a wide range of plants and crops that are essential for food production and biodiversity. The cultivation of 84% of crop species in Europe relies directly on insect pollinators, with bees playing a crucial role [1]. Klein et al. (2007) found that 87 crops, representing 70% of the 124 major crops used directly for human consumption worldwide, are reliant on pollinators [2]. Hence, the economic value of insect pollination, with bees constituting the main pollinators of plants, has been estimated to be billions of dollars annually [3,4].

Despite their ecological and economic importance, honeybee populations have been declining due to various stressors, including habitat loss, pesticide exposure, climate change, and pathogen infections [5,6]. Because honeybees forage on a wide range of crops and flowering plants, they are frequently exposed to plant protection products and other environmental contaminants [7]. Plant protection products are widely used in agriculture to protect crops, but some active substances (a.s.) have detrimental effects on non-target organisms, including bees. Several studies suggest that pesticide exposure can interfere with honeybees’ ability to forage efficiently, disrupt colony development, and, in severe cases, contribute to colony collapse [8].

While foraging, honeybees are exposed to multiple contaminants and pathogens, which they transport back to their hives, where these substances can be identified and measured in hive materials, including honey, wax, and stored pollen [9]. By analyzing the chemical composition of these products, researchers gain insights into the quality and chemical burden of the surrounding environment. For example, bees and honey have long been used to monitor heavy metal contamination in different areas, as the presence of metals can indicate environmental pollution [10,11,12]. Due to the complexity of environmental exposures, advanced analytical techniques such as mass spectrometry are increasingly used to detect and quantify chemical contaminants in honeybees and their products [13,14,15]. Additionally, chemical markers present in bee products can serve as indicators of the health and stress levels of bee colonies, providing an early warning system for environmental or pathological threats [16].

High-Resolution Mass Spectrometry (HRMS) has emerged as a powerful tool for the comprehensive analysis of environmental contaminants, metabolic changes, and biomarkers in honeybee-related studies [17]. It enables untargeted and suspect screening of xenobiotics and secondary metabolites in honey, propolis, and bee tissue [18,19,20]. Compared to traditional analytical methods, it also provides high accuracy, sensitivity, and the ability to detect unknown compounds by utilizing spectral databases and computational tools [21]. Profiling the honeybee metabolome can provide insight into physiological responses to environmental stressors and diseases [17,22,23]. To better link death incidents with analytical data interpretation, it is noted that mortality events may reflect acute poisoning. They may also be associated with sublethal effects of individual compounds or chemical mixtures. Complex exposures and mixture effects may contribute to colony-level impacts even when individual compounds do not exceed toxicological thresholds. Therefore, a wide-scope analytical profiling of honeybee samples is informative, primarily for reconstructing the exposure fingerprint and contributing to overcoming the limitations of single-compound toxicology. Similarly, it prioritizes hypotheses for follow-up experimentation needed to derive toxicological data, rather than proving in all incidents a straightforward chemical causality (unless median lethal dose (LD50) exceedances are observed). Consequently, there is a growing need for advanced analytical methods such as LC-HRMS to provide accurate and comprehensive chemical profiling of the plethora of organic chemicals that enter honeybee bodies.

In the present study, a non-targeted screening (NTS) analysis employing LC-HRMS methodology was performed to reveal the chemical context of honeybees after reported death incidents. To our knowledge, it is the first report on the application of LC-HRMS in the elucidation of the chemical profile of honeybees, despite the unambiguous use of HRMS platforms in the exploration of honeybee products in chemical markers exploitation, metabolomics, and authenticity studies [14,20,24,25]. This approach can serve as a recommended strategy for laboratories equipped with advanced equipment to integrate it into their workflow, regardless of the targeted methods, also emphasizing the potential retrospective use and interpretation of raw data. In parallel, targeted pesticide multiresidue methods were applied to provide quantitative results on pesticides that could aid in the elucidation of honeybee death incidents. By leveraging HRMS methodologies, this study seeks to contribute valuable insights into honeybee health, enabling the detection of emerging contaminants, beyond intensively investigated pesticides, with the capacity to serve as an early warning system for ecosystem health.

Given the complexity of honeybee exposure to multiple environmental contaminants, this study was designed as an exploratory investigation to address the following research questions: (i) What is the chemical exposure profile of honeybees collected after reported death incidents, beyond conventionally monitored pesticides? (ii) To what extent can non-targeted LC-HRMS complement targeted pesticide analysis in revealing additional contaminants? (iii) Does the observed co-occurrence patterns of xenobiotics provide indications of mixture and sublethal exposure scenarios relevant to honeybee health?

Overall, considering the observational nature of this study, the aim was not to establish causality but to provide a comprehensive characterization of chemical exposures associated with honeybee mortality incidents.

## 2. Materials and Methods

### 2.1. Chemicals and Reagents

Methanol (MeOH) and formic acid for liquid chromatography-mass spectrometry (LC-MS) were obtained from Merck (Darmstadt, Germany), and ammonium formate (AF) was purchased from Sigma-Aldrich (St. Louis, MO, USA). Ultrapure water (H_2_O) was produced from the SG Millipore apparatus (Singapore). All reagents and chemicals were of analytical grade. Pesticide analytical standards were obtained as mixtures from Restek Corporation (Bellefonte, PA, USA), Restek GC MIX: STD #1–9 cat.#32563–32571, Restek LC MIX: STD #1–10 cat.#31972–31981)]. Yet, a multitude of active pesticide substances were available in the analytical standards repository of the Laboratory of Pesticides’ Toxicology as individual standards and used for verification purposes. Chrysin, daidzein, sakuranetin, kaempferitrin, genistein, ursolic acid were obtained from ExtraSynthese (Genay Cedex, France), and nicotine, L-tyrosine, (−)-caryophyllene oxide, and warfarin from Sigma Aldrich (Steinheim, Germany).

### 2.2. Samples

Samples were collected from beehives following reported bee deaths in Greece. Each sample represented one mortality/incident case. Sample collection was performed either by beekeepers themselves or in collaboration with regional veterinary authorities. Samples were collected from the Peloponnese prefecture (n = 5, divided as: Argolida (n = 1), Arkadia (n = 2), and Lakonia (n = 2) regions), Crete (n = 4), Evia (n = 2), Chios (n = 2), Lesvos (n = 1) and Lefkada (n = 2) islands, Thessaly prefecture (Karditsa region, n = 2), Northern Greece (Evros prefecture, Thrace region, n = 4), Central Greece (Karpenisi region, n = 1) and Attica prefecture (n = 2). Specifically, when the researchers were contacted before sampling, they advised the sampling personnel to wear nitrile gloves (single-use) or clean cotton gloves and use precleaned metal tweezers to collect bees. In the absence of metal tweezers, the collection was instructed to be performed using a clean metal spoon. The bees (more than 50) were placed inside a glass container. Samples collected in urine caps were not incorporated in this study to avoid cross-contamination in the LC-HRMS analysis because of the chemical components of the plastic urine caps. After collecting the samples, if the collection area was near the laboratory (distance of 2–3 h), they were dispatched the same day in a cool box containing two ice packs. Otherwise, samples were stored at −20 °C and sent to the laboratory the next day, following the same procedure. Upon arrival, if not treated the same day, the samples were stored at −20 °C and treated within 2 days. As controls and samples for fortification experiments and baseline comparison, honeybee samples from the laboratory repository, previously assessed for pesticide residues (blank for pesticides within the targeted scope of the method), were used to facilitate accurate determination of positive findings.

### 2.3. Sample Preparation for UHPLC-HRMS/MS Analysis

The extraction and clean-up of the honeybee extracts were principally based on a modified QuEChERS method, previously published by our group [26,27] for LC and GC-MS/MS analysis, excluding the use of n-hexane in the first step (see Supplementary Materials respective section). To achieve the desired mass of bees, approximately 10–12 honeybees (average weight of 0.1 g per bee) were weighed. Briefly, after homogenization of 1 g of bees in water and the addition of acetonitrile, the homogenate was partitioned (phase-separated) using MgSO_4_ and sodium acetate (acting as a buffering agent). Then, after centrifugation, the supernatant layer was cleaned up using PSA and MgSO_4_ and split into two parts for LC and GC analysis.

### 2.4. UHPLC-HRMS/MS Analysis

The chemical profiling of honeybee extracts was determined using a Dionex Ultimate 3000 UHPLC system (Thermo Scientific, Karlsruhe, Germany) with a Q-Exactive Orbitrap mass spectrometer (Thermo Scientific, Karlsruhe, Germany). A Fortis (Cheshire, UK) Speedcore C18 reverse-phase column (2.6 μm, 100 × 2.1 mm) was employed for UHPLC separation, with the column oven maintained at 25 °C. The mobile phases were MeOH (B) and water (A) with 0.1% formic acid and 5 mM ammonium formate for negative and positive ion mode, respectively. The chromatographic separation was carried out over a 24 min analysis using the following gradient elution program for the mobile phases: T = 0 min, 2%B; T = 2 min, 2%B; T = 17 min, 100%B, T = 21 min, 100%B, T = 21.2 min, 2%B, T = 24 min, 2%B, with a flow rate of 0.22 mL/min, and the injection volume was 5 μL. The MS1 system settings were *m*/*z* = 120–1000 (mass range), resolution = 70,000, and AGC target = 5 × 105, and the MS2 system settings were resolution = 17,500, AGC target = 1 × 105, and (N)CE = 40, 60, and 100. The analysis was performed in negative and positive ionization mode, with a sheath gas flow rate of 40 arbitrary units, auxiliary gas flow rate of 5 arbitrary units, spray voltages of 2.7 kV (for negative ionization) and 4 kV (for positive ionization), capillary temperature of 350 °C, S-lens RF level of 50, and auxiliary gas heater temperature of 50 °C. Data-dependent acquisition was applied allowing for MS/MS fragmentation of the six most intense ions of every peak, applying a 5 s dynamic exclusion. During the analysis, solvent blanks, procedural blanks, and instrument blanks were included, along with duplicate injections and calibration checks, to ensure reliability of detection and to minimize background contamination.

### 2.5. Data Post-Processing Analysis

Raw data were exported to Compound Discoverer v3.3.3 (Thermo Fisher Scientific Inc., San Jose, CA, USA) for peak detection, deconvolution, deisotoping, and composition prediction procedures. The parameters introduced in the workflow analysis were to detect compounds with mass tolerance of 3 ppm and min peak area intensity 106; merge features with mass tolerance of 3 ppm and RT tolerance of ±0.2 min; group compounds with mass tolerance of 3 ppm and RT tolerance of ±0.2 min; align peaks to true; and mark background compounds with S/N threshold of 1.5 and max sample/blank ratio of 5. Compound tentative identification relied on spectral library searches and in silico fragmentation tools. Specifically, for the identification of the compounds, the mzCloud (https://www.mzcloud.org, accessed on 30 June 2024) and the NIST/EPA/NIH Mass Spectra Library 2020 databases were used applying m/z tolerance of 3 ppm, taking into consideration the isotope distribution similarity and MS/MS fragmentation pattern. Compound identification confidence levels were assigned to the criteria described by a recent work of Alygizakis et al. [28], also considering the earlier work by Schymanski and colleagues [29]. For the compounds for which there was no record, the MetFrag in silico fragmentation tool [30] was used, applying 3 ppm search tolerance and 0.001 mass deviation to match generated fragments against MS/MS peaks. For the MetFrag workflow, the candidate structures were retrieved from the Kegg database. For the compounds that overlapped with the targeted pesticide analysis, confirmation was performed using authentic reference standards. In these cases, retention time alignment together with matching MS/MS fragmentation patterns against the reference compounds ensured confident structural identification. For the tentative identification of Per- and Polyfluoroalkyl Substances (PFAS) in honeybee extracts, a dedicated workflow for unknown PFAS identification using database searches was applied within Compound Discoverer v3.3.3. In the Compound Class Scoring node, the databases PFAS Fine Structure Fragment Library and PFAS General from the FluoroMatch Suite were used, applying mass accuracy tolerances of ±5 ppm (high) and ±10 ppm (low), respectively. The signal-to-noise (S/N) threshold was set to 1. In the Search Mass Lists node, the following suspect lists were queried, all with a mass tolerance of 5 ppm: PFAS_Neg, PFAS_NIST, PFAS_suspectDB_Duke, and the PFASSTRUCT-2022-04-20 chemical list. For MS/MS spectral interpretation, the MetFrag web tool was employed to compare experimental fragmentation spectra with theoretical spectra generated via in silico fragmentation. The PubChem_OECDPFAS_largerPFASparts_20250125 database was used as a compound source, which contains larger structural PFAS subunits aligned with recent OECD PFAS classifications. A search tolerance of 3 ppm and a matching deviation of 0.001 Da were applied.

To this end, the LC-HRMS analysis was applied as a non-targeted screening tool to qualitatively explore the chemical composition of the samples and identify unexpected or emerging contaminants; therefore, the workflow was not intended for compound-specific quantification, which, for pesticides, was addressed separately by validated LC, GC-MS/MS methods.

### 2.6. LC, GC-MS/MS Analysis

LC/MSMS analysis was conducted on a Shimadzu 8060NX triple quadrupole system (Shimadzu Corporation, Kyoto, Japan). Chromatographic conditions were the same as published in previous work of our group. Mass spectrometer functioned in the electrospray ionization (ESI) mode, with MS parameters optimized for the quantification and qualification ions, the respective energies Q1 pre bias (V), collision energy (CE) and Q3 pre bias (V). Standard solutions (at 100 ng/mL) were used for the optimization. GC-MS/MS analysis was performed in a Shimadzu GCMS-TQ8040 NX triple quadrupole (Shimadzu Corporation, Kyoto, Japan). Data acquisition and processing were conducted by LabSolutions GCMS solution software, version 4.52. Samples were injected using a PTV injector inlet in splitless mode, through a Shimadzu ultra-inert inlet liner with glass wool frit (Shimadzu Corporation, Kyoto, Japan). The injection volume was 2 µL. The injector temperature was kept at 250 °C. A MEGA 5-HT (MEGA S.r.l., Legnano, Italy) column (30 m length × 0.25 mm i.d. × 0.25 µm film thickness) was used.

The oven temperature program was as follows: Starting from 50 °C (held for 1 min), increased to 100 °C at 20 °C/min, ramped linearly to 200 °C at 5 °C/min, increased to 280 °C at 5 °C/min (held for 5 min), finally reaching 300 °C with a rate of 5 °C/min and held at this temperature for 10 min. The total running time was 58.5 min. The instrument worked at a constant flow of 1.69 mL/min, using the (multiple reaction monitoring) MRM mode, acquiring the transitions in a ±0.2 min window from the retention time of each analyte. Helium (99.999% purity) was used as the carrier gas and argon (99.999% purity) as the collision gas. The transfer line and the ion source—operated in electron ionization— were maintained at 250 °C and 230 °C, respectively. The detector voltage was set at 0.6 kV (relative to the tuning result). The quadrupole analyzer temperature was fixed at 150 °C. The solvent delay (cut time) was 1.5 min. MRM conditions for LC, GC-MS/MS methods are presented in the Supplementary Materials (Excel file).

### 2.7. Analytical Method Validation

Validation of the analytical method was grounded on the SANTE guideline [31]. For 146 active substances (parent compounds and metabolites) not included in the previous works of our group (in bees [26,27,32]), a thorough validation of the method was performed (at LOQ, 10× LOQ, and 100× LOQ). For 38 substances (previously dealt for bees), sensitivity was improved, and updated validation metrics are also depicted. In total, more than 250 a.s. were monitored (an indicative TIC MRM chromatogram is presented in Figure S1; for validation of the method see the Supplementary Materials). To avoid false-positive results, emphasis was given to the consistency of the ion ratio of the MRM transitions in the actual samples (shown in Table S1, ±30% relative) to those of the spiked and analytical standard solutions.

### 2.8. In Silico Calculations for Toxicological Assessment

For ecotoxicity in silico explorations, the open-access online resource “cropCSM: identifying safe and potent herbicides” [33] was used by incorporating the simplified molecular-input line-entry system (SMILES) string for the studied compounds.

### 2.9. Honeybee Health Risk Assessment

To assess the risk of pesticides to bees, the European Food Safety Authority (EFSA) bee guidance document and its revision were regarded [34,35] along with the recommendations by the United States Environmental Protection Agency (US-EPA) [36]. Consequently, the concentration-based risk quotient approach (as RQ bees) was implemented by dividing the actual pesticide residues detected in honeybees by their LD50 values (oral and contact). The RQbees threshold-limit of concern value was set to 0.1, assuming a one-day consumption based on the EFSA recommendation [34], and for acute lethality was established by US-EPA at 0.4 [36]. According to this approach, RQ values exceeding 0.1 indicate a potential risk for honeybees, while RQ values above 0.4 indicate a high risk of acute toxicity.

## 3. Results and Discussion

**Figure 1:**
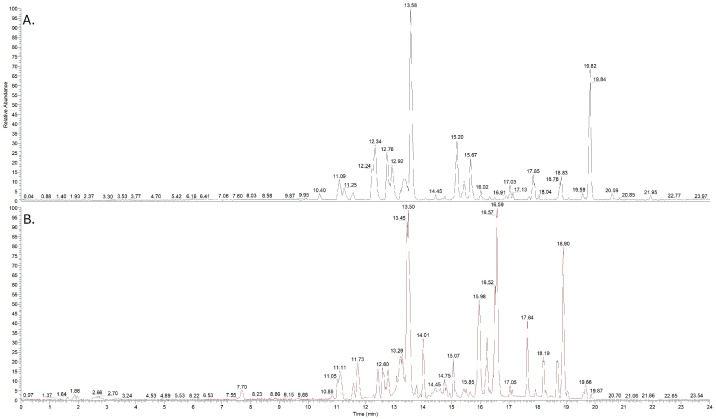
Representative chromatograms of honeybee extracts after UHPLC-HRMS analyses in positive (**A**) and negative (**B**) ionization modes.

An NTS workflow based on HRMS was employed to investigate chemical exposure in honeybees. This approach enabled the comprehensive detection and characterization of a wide range of chemical compounds (more than 50; a series of indicative chromatograms are presented in Figure 1 and in Supplementary Materials, Figures S2–S26). Utilizing the sensitivity and resolution of HRMS, we identified potential contaminants and bioactive substances, enabling characterization of the chemical profile of honeybee extracts. Table 1 summarizes the detected compounds, including their monoisotopic mass, MS/MS fragment ions, and molecular formula. As this part of the study employed a non-targeted exploratory screening approach, statistical comparative analysis was not within its intended scope. Nevertheless, the detection frequency across the analyzed samples, expressed as % percentage, was calculated (Table 1). Similarly, the random character and the limited number of incidents restricted any statistical attempt to correlate geographic origin and sources of contaminants.

The HRMS analysis not only disclosed the presence of chemicals apart from pesticides but also revealed the presence of additional pesticide a.s. that were not within the scope of the targeted LC-MS/MS methods.

### 3.1. Pesticides Detection

It is acknowledged that use of pesticides in agricultural settings can affect bee populations by altering the availability of foraging plants or by contaminating nectar and pollen [37]. In this work, both targeted and untargeted chemical analysis disclosed the presence of 15 pesticide a.s. and one hydrolysis product of carbaryl (1-naphthol, detected in the same sample with carbaryl). Specifically, HRMS along with an in silico fragmentation (an approach with a multitude of applications [38,39]) unveiled the a.s., propoxur (insecticide), fenobucarb (insecticide), bifenazate (insecticide) and acequinocyl (acaricide) (that were not within the scope of the targeted method). The latter can be valuable in cases of bee death incidents, as the disclosure of additional compounds with relatively high signals (and subsequent abundance) may facilitate their interpretation. In our case, due to the relatively low signal and high LD50 values of the tentatively identified pesticides, quantification (or semi-quantification) of these substances was not pursued. Specifically, due to the relatively low signal intensity and lack of reference standards, quantitative or semi-quantitative determination of these tentatively identified compounds was not performed.

**Figure 2:**
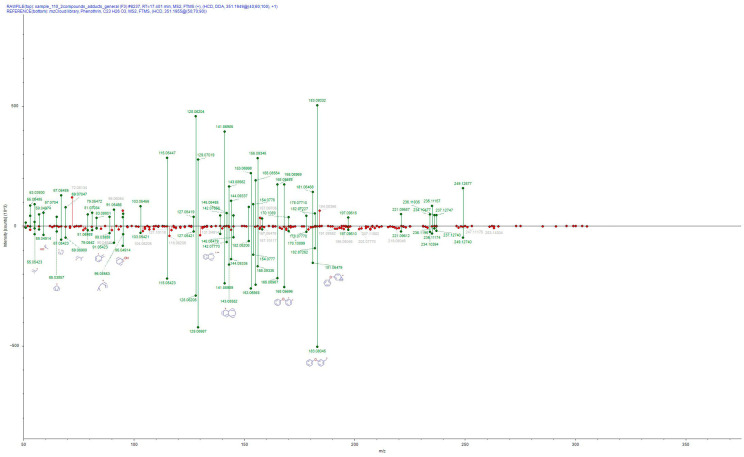
Phenothrin annotation based on MS/MS fragmentation pattern using mzCloud database. The top panel is the MS/MS pattern of the honeybee extract, whereas the bottom panel is the MS/MS pattern of phenothrin from the mzCloud database.

In the same context, LC, GC-MS/MS revealed the presence of additional pesticide a.s. (see Table 2), some of them not identified by LC-HRMS, possibly due to their better amenability to gas chromatography, and sensitivity features of the HRMS system compared to the triple quadrupole. Among these compounds were the pyrethroids λ-cyhalothrin, bifenthrin and permethrin. Physicochemical properties of pyrethroids have been long known to favor GC determination due to enhancement in sensitivity and specificity [40]. For tau-fluvalinate, phenothrin (phenothrin’s annotation is shown in Figure 2 and MRM chromatogram in Figure S27), and propiconazole, both LC-HRMS and LC, GC-MS/MS verified their presence (a marginal-low identification score HRMS identification of imidacloprid was attributed to its low concentration that was not an obstacle in triple quadrupole analysis). Tau-fluvalinate was the predominant compound among positive findings (n = 3), followed by fluopyram, phenothrin, and propiconazole. Overall, pesticide detections were not uniform across samples, and no single active substance emerged as a consistent marker across all mortality cases. Instead, selected samples exhibited combinations of insecticides and fungicides, underscoring the heterogeneity of exposure scenarios potentially associated with reported honeybee death incidents.

From a method validation perspective, targeted chemical analysis validation criteria complied with the guideline’s requirements [31]. Sufficient LOQs were demonstrated in the range of 0.0005 to 0.005 mg/kg (related to concentrations provoking sublethal effects) with overall recoveries fluctuating from 63 to 119%, exhibiting acceptable RSD% not higher than 17% and moderate matrix effects for most of the analytes (for detailed validation data see Supplementary Materials, respective paragraph and Table S1). Precision, as portrayed by the repeatability and reproducibility RSD% values at the three concentration levels, was satisfactory (≤20%, Table S1).

### 3.2. Neuroactive Compounds and Bee Behavior

**Figure 3:**
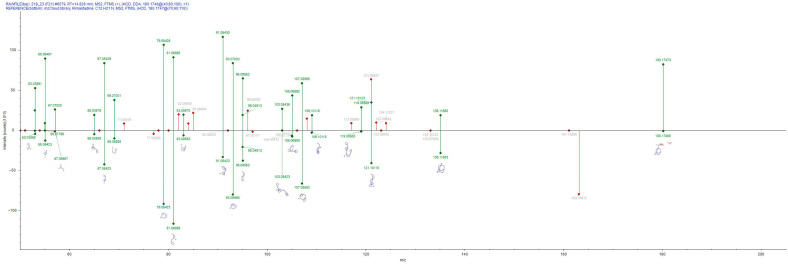
Rimantadine annotation based on MS/MS fragmentation pattern using mzCloud database. The top panel is the MS/MS pattern of the bee extract, whereas the bottom panel is the MS/MS pattern of rimantadine from the mzCloud database.

Compounds such as nicotine, gabapentin and rimantadine (annotation in Figure 3) affect neural pathways and cognitive functions in honeybees. More specifically, the presence of nicotine in bee extracts may indicate exposure to environmental pollutants, such as nicotine-containing pesticides [41]. However, nicotine is also naturally found in some plant nectars [42]. Additionally, a decrease in the consumption of nicotine-containing food may influence response levels during learning and memory assessments [43]. Interestingly, rimantadine’s identification, though not anticipated (is an oral antiviral drug), is partly in line with the work of Wang and coworkers, who detected rimantadine with a substantially low rate (0.18%) in honey [44].

### 3.3. Tentative Identification of PFAS in Honeybee Extracts

In silico prediction tools are known for their capacity to identify xenobiotic metabolites [45]. In this work, in silico fragmentation along with NTS suggested the presence of 15 PFAS (Table S2). PFAS were specifically targeted due to their persistence, bioaccumulation potential and emerging relevance as environmental contaminants of concern. However, their structural diversity combined with the limited availability of reference standards and mass spectrometry data constrain confidence identification. For this reason, PFAS-related signals detected in this study are interpreted conservatively at the level of fluorinated formula candidates rather than specific chemical structure. Notably, neither of these compounds belongs to the 4 PFAS elaborated by EFSA and for which a tolerable weekly intake has been established, nor to the extended list currently being investigated in various matrices [46,47]. In addition, more than 35 a.s. that are currently approved have been reported to fall under specific structural definitions of PFAS [48] (in this work, bifenthrin, λ-cyhalothrin, and tau-fluvalinate); however, reports indicate that despite concerns about PFAS, these chemicals may still be present in pesticide formulations or may originate from leaching of fluorinated containers [49].

From an ecotoxicology point of view, the open-access cropCSM online resource, designed for the toxicological assessment of small molecules (driven by herbicides), including honeybee toxicity (as a categorical yes/no), was pilot-contemplated for the 15 PFAS. Despite the limitations of the cropCSM tool (not designed for a broad range of chemicals) and the resulting uncertainty in predictions (which do not constitute concrete evidence of adverse effects in honeybees), assigning potential chemical structures identified two plausible PFAS as potentially toxic to honeybees. More specifically, for both compounds (2 and 14), the common alicyclic hydrocarbon system (in Figure S28, see their potential chemical structures) might play a role in the estimated toxicity to bees. The literature designated that exposure of honeybees to environmentally relevant PFOS concentrations led to bee mortality and hampered brood development [50]. In the same context, greenhouse experiments using *Cannabis sativa* L. as a medium to remediate PFAS-contaminated soil verified PFAS uptake by pollen [51] and the potential exposure of bees to these chemicals via pollen consumption. Consequently, the tentative identification of less-known PFAS analogs should not be overlooked, as they may affect bees’ physiological behavior, although no direct contribution to mortality can be demonstrated within the scope of this study.

### 3.4. Other Environmental Contaminants

Industrial chemicals are increasingly being detected in honeybee habitats [19] due to agricultural and industrial activities. A recent publication documented their prevalence in agricultural soils as a result of conventional and biodegradable plastic mulches [52], while bee pollen is also a matrix with reported occurrence of such chemicals [53]. Among phthalates, both dimethyl phthalate and diisobutyl phthalate were tentatively identified. Dewaele and Cuvillier confirmed that bumblebees exhibited high phthalate contamination, with gene expression analysis revealing endocrine-disrupting effects on these wild bees [54]. Interestingly, the identification of triisopropanolamine might postulate the use of acidic herbicides containing it as a neutralizing agent [55], without excluding other uses (e.g., cosmetics) due to its emulsifying potential [56].

### 3.5. Plant-Derived Alkaloids and Their Effects

Scopolamine, a muscarinic antagonist, has been shown to impair bees’ ability to recognize nestmates, leading to increased aggression toward both familiar and unfamiliar individuals, highlighting the importance of muscarinic signaling in olfactory-based social recognition [57]. Moreover, a study by Sculfort et al. examined how certain floral compounds, including scopolamine, affect buff-tailed bumblebees (*Bombus terrestris*) [58]. According to their results, at the individual level, bumble bees consuming the 50% scopolamine diet exhibited notable digestive tract damage, indicating that while colonies may appear resilient, individual bees can suffer adverse effects that could influence overall colony health.

### 3.6. Cannabinoids and Their Potential Effects

**Figure 4:**
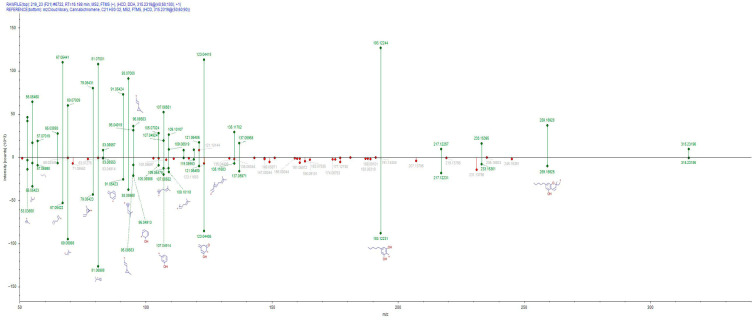
Cannabichromene annotation based on MS/MS fragmentation pattern using mzCloud database. The top panel is the MS/MS pattern of the honeybee extract, whereas the bottom panel is the MS/MS pattern of Cannabichromene from the mzCloud database.

HRMS revealed the presence of two cannabinoids—cannabichromene (annotation depicted in Figure 4) and cannabidiolic acid—in honeybee-derived extracts. Experimental supplementation with hemp extract has been shown to increase total protein levels and enhance protease inhibitors’ activity, which are essential components of the insect innate immune response [59]. From another viewpoint, though cannabis is wind-pollinated, the confirmation of cannabinoids in honeybees may indicate that they collect pollen from cannabis [60,61].

### 3.7. Other Bioactive Compounds

Secondary metabolites present in floral resources can influence key aspects of honeybee health, including immunity, detoxification, gut microbiota, and behavior [62]. Protective effects of flavonoids such as rutin and other bioactive compounds were reported when bees were exposed to the highly toxic insecticide fipronil [63]. In the study by Li et al. [64], metabolomic analyses revealed that royal jelly produced by bees fed soybean meal-based diets contained elevated levels of the isoflavones daidzein and genistein (detected in three of the bee samples evaluated herein). This finding is of particular interest since royal jelly constitutes a pivotal food for honeybee larvae. The complex flavonoid glycoside kaempferitrin, found in certain medicinal plants visited by bees, exhibits free radical scavenging capacity and may assist in mitigating oxidative damage, especially in bees exposed to pesticide residues [65]. Finally, the identification of 1-naphthalene acetic acid (a plant growth regulator) is logical, given its documented use in agriculture and subsequent attention paid in its risk assessment by EFSA [66].

### 3.8. Fatty Acids and Their Role in Honeybee Physiology

Dietary supplementation with arachidonic acid (tentatively identified in this work) has been associated with enhanced larval growth and improved immune parameters in *Apis mellifera ligustica*, as well as enhanced overall survival and resilience to pathogenic stress [67]. Εthyl oleate (EO), another fatty acid ester detected in bees’ extracts, is a primer pheromone that delays the onset of foraging behavior in younger worker bees, thus helping the colony maintain a balanced distribution of tasks across age groups. It should also be noted that these compounds are well-known endogenous metabolites in honeybees, involved in physiological and signaling processes. However, their presence may also be influenced by environmental or dietary sources, and their origin cannot be clearly determined within the scope of this study.

### 3.9. Volatile (And Semi-Volatiles) Organic Compounds and Bee Attraction

Several compounds, such as acetophenone (see Figure S5 for its annotation), methyl eugenol, and trans-cinnamaldehyde, play a central role in plant–pollinator interactions. Floral scents are crucial for guiding bees to nectar and pollen sources, thereby aiding in efficient pollination [68]. Methyl eugenol, a phenylpropanoid found in many plant species, is known for its ability to attract certain pollinators [69]. Eugenol has been shown to exhibit insecticidal properties, which could be beneficial for bees in controlling harmful pests [70]. However, at higher concentrations, these compounds could pose toxicity risks to bees [71].

### 3.10. Honeybee Health Risk Assessment

Among the bee death incidents investigated in this study, we did not observe exceedances of the LD50 values. Nevertheless, there was one case in which imidacloprid was detected at a concentration within a range reported to provoke sublethal effects on bees [72]. In the same sample, the pyrethroids permethrin, fungicide propiconazole, insecticide carbaryl, stylopine and triphenyl phosphate were also detected, suggesting potential mixture effects on honeybees and standalone sublethal effects based on the detected levels (indicatively see [73]). Notably, in this sample, 1-naphthol, a metabolite of carbaryl [74] was also detected via HRMS analysis, corroborating the analytical capability of the HRMS methodology. It is also noteworthy that ergosterol biosynthesis-inhibiting fungicides, such as propiconazole, act synergistically, substantially lowering the LD50 of the pyrethroid λ-cyhalothrin [75]. Gil and coworkers demonstrated that exposure of bumblebees to field-realistic levels of neonicotinoids and pyrethroids impacted foraging behavior and increased worker mortality, leading to notable decreases in brood development and colony well-being [8]. Wang and coworkers showed that thiamethoxam in combination with pyrethroids (exemplified by λ-cyhalothrin) exerted synergistic toxic effects on *Apis mellifera ligustica* honeybees [76]. In the same context, mixtures (ternary or more) containing neonicotinoids and pyrethroids exhibited both synergistic and additive effects on bees [64]. Consequently, analogous effects cannot be excluded in the presented study.

The broadly used fungicides boscalid and fluopyram were also detected at levels substantially lower than their LD50 values. For boscalid, higher concentrations (than those herein presented), but environmentally relevant, were reported to affect the reproduction of immature queen bees [77]. Fluopyram in combination with another fungicide, tebuconazole, induces oxidative stress and affects honeybee survival [78], but at much higher concentrations compared to those herein evidenced.

To move a step further from “simplistic” comparisons and literature quotation, the RQs and the respective “no concern level, NCL” introduced by EFSA [35] were calculated for the detected active substances and are portrayed in Table 3. NCLs correspond to exposure levels in bees (mg/kg bw) that are not expected to affect their foraging behavior. As actual exposure levels for both oral and contact ratio calculations, the maximum concentrations of the respective a.s. in bees were considered. The latter was decided due to the low number of detections (n = 2) for relatively toxic insecticides (in bold, Table 3); hence, the distribution of concentrations (i.e., the 90th percentile) cannot be applied for risk assessment calculations and comparisons with the RQ and NCL thresholds. The RQ values for most substances were far below the threshold values of 0.1 and 0.4 set by EFSA and US-EPA, respectively, except for permethrin and phenothrin, which surpassed them, indicating a potential risk.

To provide further insight into sublethal assessment, the NCLs (oral and contact) of the detected pesticides were determined by dividing the corresponding LD50 values by 50, and the maximum concentrations exceeding this value are juxtaposed. For pyrethroids and imidacloprid, maximum detected concentrations exceed NCLs, corroborating the RQ assessment for pyrethroids and, in parallel, supporting the documented sublethal effects of these pesticide categories. Nevertheless, in risk calculations and subsequent conclusions, the uncertainty arising from potential chemicals’ decomposition in the field before sampling should be considered, and in some instances, additional information can be pursued from beekeepers and veterinary authorities. In the Mediterranean area in particular, beekeeping is largely nomadic (beekeepers are not always in proximity to their apiaries), and several days can pass between the incident and sampling.

An informative meta-analysis on binary mixture toxicity to bees was published by Carnesecchi et al. (2019) in which, in addition to dominant pesticides, other chemicals are implicated in such effects [79]. Among them are the flavonoid quercetin and the antibiotics oxytetracycline, ivermectin and tylosin. Interestingly, tau-fluvalinate, detected in this work, is among the pesticides exhibiting combined toxicity with a variety of chemicals, including pharmaceuticals (i.e., salicylic acid, xanthotoxin), antibiotics (such as fumagillin, tylosine, oxytetracycline), and NPCs (such as quercetin) [79]. In this sense, it is characteristic that in the presented work the sample most contaminated with tau-fluvalinate simultaneously contained 12 chemicals (namely trans-cinnamaldehyde, (−)-caryophyllene oxide, chrysin, diisobutyl phthalate, ethyl oleate, triphenyl phosphate, cannabidiolic acid, dimethyl phthalate, carbaryl, oxabetrinil, warfarin and betulinic acid methyl ester). The most prevalent chemical combination (observed in 25% of samples) was ethyl oleate with (−)-caryophyllene oxide. Other common patterns were observed in pairs of two or more samples. The first consisted of genistein, ethyl oleate, and triphenyl phosphate, while other binary combinations were (−)-caryophyllene oxide with isobornyl methacrylate (and with chrysin).

Consequently, even if different co-occurring chemicals as those described in the literature are evidenced, the detection pattern can provide “realistic” assumptions for such combined (additive, synergistic) toxicity effects, apart from the risk due to exposure to a single pesticide active substance. In the same context, not-approved pesticides are still detected (in this work, 53% of pesticides detected) in the environment, a fact that should be dealt with caution by the beekeepers, veterinary and related authorities. Compared to previous work by our group [27,32], the positive honeybee samples in pesticides are decreased, which underpins the implementation of risk management measures in the specific beehive areas, though the pilot character of this study and the restricted number of samples cannot be used for generic conclusions. With regard to the other detected chemicals (apart from pesticides), their ecological relevance cannot be readily assessed due to the lack of a) quantitative (concentration) data (outside the scope of the presented work) and b) toxicological endpoint values (e.g., LD50).

Based on the findings presented, honeybees are exposed to a chemical arsenal comprising a multitude of contaminants and other bioactive compounds. Importantly, the available data does not allow causal attribution of mortality to specific chemicals. Instead, they indicate that honeybees are exposed to complex mixtures of contaminants and bioactive compounds, highlighting the limitations of single-compound risk assessment and suggesting that the concomitant presence of potentially beneficial substances may contribute to resilience against xenobiotic stress. Selected cases combined neonicotinoid/pyrethroid/fungicide signatures together with other contaminants, a pattern repeatedly discussed in the mixture-toxicity literature as potentially relevant for behavior, toxicological effects, detoxification burden, and colony performance. As indicated above, a partial limitation of the present study is the lack of a biological association with the results, which limits the direct ecotoxicological interpretation of the findings. Future studies should integrate chemical analysis with effect-based approaches, including behavioral, physiological, or molecular biomarkers, to better link exposure patterns with biological responses in honeybees.

Overall, while the present dataset lacks effect measures and therefore cannot test these hypotheses, it provides an evidence-based chemical exposure map to prioritize targeted mixture experiments and effect-based monitoring in future incident investigations.

## 4. Conclusions

Honeybees are critical to the functioning of ecosystems and agriculture. Therefore, the chemical investigation of mortality incidents is essential for understanding the impact of environmental stressors on their health, behavior, and survival. In this work, LC-HRMS non-targeted screening of 25 honeybee extracts identified more than 50 chemicals across multiple compound classes, including industrial chemicals/plasticizers, pharmaceuticals, and fluorinated PFAS candidates not included in the EFSA “PFAS-4” list, expanding knowledge beyond pesticides. In parallel, LC, GC-MS/MS targeted methods provided quantitative insights into pesticides, with quantified concentrations not exceeding the acute LD50 thresholds. The produced dataset, though highly informative, does not allow a straightforward causal attribution of mortality to any single compound or combination. Nevertheless, the co-occurrence patterns (i.e., pesticides plus other xenobiotics and bioactives) underscore the potential importance of mixture effects and sublethal exposures which may contribute to honeybee death incidents.

Moreover, the relatively limited sample size and the absence of a harmonized–stratified sampling design limit the possibility of statistical comparisons and generalization of the findings; therefore, the results should be interpreted as indicative of exposure patterns rather than representative of broader honeybee population-level trends.

Future work should integrate chemical profiles with effect-based endpoints (behavioral/physiological biomarkers), bee-relevant mixture experiments, and harmonized confidence reporting for NTS to strengthen inference of incident causality.

Finally, research directions may include the integration of HRMS data with advanced data analysis approaches to deepen understanding of honeybee health and conservation strategies, aiming to develop early-warning systems and effective risk management.

## Figures and Tables

**Table 1:** Contaminants and bioactive substances detected on honeybee extracts, employing HRMS analysis and a non-targeted screening (NTS) workflow.

No.	Compound Name	Monoisotopic Mass (Da) Experimental_tR (min)	Molecular Formula	MS/MS Fragment Ions (*m*/*z*)	Identification Level (from 1 to 5 According to the IP Scores)	Annot.DeltaMass (ppm)	Frequency_(%)	Chemical Class	Use
1	Acetophenone	120.0576_12.83	C_8_H_8_O	121.065/105.070/103.054	3	0.71	8.33	Benzene derivative	Synthetic precursor
2	Skatole	131.0736_10.44	C_9_H_9_N	132.081/117.057	3	0.68	8.33	Indole derivative	Mammalian metabolite
3	trans-Cinnamaldehyde	132.0576_15.12	C_9_H_8_O	133.065/115.054/105.070	3	0.41	12.5	Benzene derivative	Natural product constituent (NPC)
4	1-Naphthol	144.0577_14.97	C_10_H_8_O	117.070/115.054/145.065	3	1.15	4.17	Naphthalene metabolite	Coloring agent
5	1,5-Isoquinolinediol	161.0477_10.02	C_9_H_7_NO_2_	116.050/144.044/162.055	3	0.14	4.17	Isoquinoline derivative	Poly(ADP-ribose) polymerase inhibitor
6	Nicotine ^a^	162.1159_12.46	C_10_H_14_N_2_	106.065/117.057/132.081	1	1.01	4.17	Alkaloid	Stimulant
7	Eugenol	164.0838_20.04	C_10_H_12_O_2_	119.085/137.096/107.049	4	0.39	12.5	Phenol	Aromatic/flavoring agent
8	Gabapentin	171.126_12.07	C_9_H_17_NO_2_	154.123/109.101/119.086	3	0.19	12.5	Cyclohexane derivative	Pharmaceutical
9	Methyl eugenol	178.0994_12.36	C_11_H_14_O_2_	121.065/105.070/1037.096	4	0.3	12.5	Phenol derivative	Flavoring agent
10	Rimantadine	179.1675_14.83	C_12_H_21_N	121.101/107.086/135.117	3	0.46	4.17	Alkylamine	Pharmaceutical
11	2-Amino-6-methylmercaptopurine	181.0423_11.78	C_6_H_7_N_5_S	182.050/134.046/107.035	3	0.46	12.5	Amino acid metabolite	
12	L-Tyrosine ^a^	181.074_4.48	C_9_H_11_NO_3_	136.076/123.044/119.049	1	0.45	25	Amino acid	
13	1-Naphthaleneacetic acid	186.0682_14.65	C_12_H_10_O_2_	103.054/115.054/131.086	4	0.82	4.17	Naphthalene derivative	Plant growth regulator
14	Triisopropanolamine	191.1523_10.17	C_9_H_21_NO_3_	174.149/192.160/156.138	3	0.67	12.5	Amine	Industrial applications
15	Dimethyl phthalate	194.058_15.21	C_10_H_10_O_4_	117.033/145.028/177.055	4	0.64	20.83	Phthalates	Plasticizer
16	D-Panthenol	205.1316_9.91	C_9_H_19_NO_4_	188.128/170.118/102.055	3	0.98	4.17	Butanamide derivative	Cosmetic
17	Fenobucarb ^a^	207.1261_12.83	C_12_H_17_NO_2_	121.065/103.054/95.049	1	0.5	12.5	Carbamate derivative	Insecticide
18	Propoxur	209.1053_11.67	C_11_H_15_NO_3_	151.075/119.049/107.049	4	0.55	8.33	Carbamate derivative	Insecticide
19	Syringate	212.0687_13.31	C_10_H_12_O_5_	181.049/154.062/139.039	3	0.84	4.17	Benzoic acid derivative	NPC
20	N-Butylbenzenesulfonamide	213.0826_14.22	C_10_H_15_NO_2_S	141.000/158.027/105.045	3	0.95	4.17	Sulfonamide	Plasticizer
21	Carbaryl ^a^	201.0791_11.18	C_12_H_11_NO_2_	160.076/115.054/132.081	1	0.81	4.17	Carbamate derivative	Pesticide
22	(−)-Caryophyllene oxide ^a^	220.1828_15.66	C_15_H_24_O	109.101/119.085/105.070	1	0.46	25	Sesquiterpene	Non-sensitizing agent
23	Isobornyl methacrylate	222.1621_14.32	C_14_H_22_O_2_	121.101/107.086/105.070	3	0.51	8.33	Acrylic ester monomer	Solvent
24	Icaridin	229.1679_14.74	C_12_H_23_NO_3_	130.123/112.112/128.071	3	0.49	8.33	Piperidine derivative	Insect repellent
25	Dimethyl sebacate	230.1518_13.7	C_12_H_22_O_4_	121.101/139.112/107.086	3	0.01	4.17	Dicarboxylic acid derivative	Plasticizer
26	Oxabetrinil	232.08491_11.41	C_12_H_12_N_2_O_3_	174.055/146.060/118.065	4	0.5	12.5	Benzene derivative	Herbicide safener
27	Chrysin ^a^	254.0579_14.21	C_15_H_10_O_4_	153.018/103.054/128.062	1	−0.05	16.67	Flavonoid	NPC
28	Daidzein ^a^	254.0579_13.57	C_15_H_10_O_4_	199.075/181.065/137.023	1	−0.1	8.33	Isoflavone	Phytoestrogen
29	Genistein ^a^	270.0529_13.94	C_15_H_10_O_5_	153.018/215.070/243.065	1	0.21	12.5	Isoflavone	Phytoestrogen
30	Diisobutylphthalate	278.1519_18.56	C_16_H_22_O_4_	149.023/121.028	3	0.26	12.5	Phthalates	Plasticizer
31	Sakuranetin ^a^	286.0841_16.25	C_16_H_14_O_5_	167.034/147.044/119.049	1	0.02	8.33	Flavonoid	NPC
32	Fenbendazole	299.0728_15.21	C_15_H_13_N_3_O_2_S	159.043/268.054/131.048	3	−0.26	4.17	Benzimidazole	Pharmaceutical
33	Bifenazate	300.1474_11.94	C_17_H_20_N_2_O_3_	121.065/136.076/119.049	4	0.08	4.17	Carbazate	Insecticide
34	Scopolamine	303.1471_12.98	C_17_H_21_NO_4_	138.091/103.054/156.102	3	0.08	4.17	Alkaloid	Pharmaceutical
35	Arachidonic acid	304.2403_18.10	C_20_H_32_O_2_	105.070/121.101/133.101	3	0.35	12.5	Fatty acid	Cell membrane component
36	Warfarin ^a^	308.105_15.73	C_19_H_16_O_4_	205.050/177.055/149.060	1	0.45	12.5	Coumarin derivative	Anticoagulant
37	Ethyl oleate	310.2873_20.09	C_20_H_38_O_2_	121.101/135.117/107.086	3	0.43	33.33	Fatty acid ester	Additive
38	Cannabichromene ^a^	314.2247_16.19	C_21_H_30_O_2_	193.122/123.044/259.169	1	0.44	4.17	Cannabinoid	NPC
39	Stylopine	323.1158_15.82	C_19_H_17_NO_4_	176.071/149.059/119.049	3	0.04	4.17	Alkaloid	NPC
40	Triphenyl phosphate ^a^	326.0708_21.92	C_18_H_15_O_4_P	152.062/233.036/251.047	1	−0.01	32.00	Organophosphorus agent	Flame retardant
41	Propiconazole ^a^	341.06981_15.58	C_15_H_17_C_l2_N_3_O_2_	158.976/172.955	1	0.08	4.17	Triazoles	Fungicide
42	Cannabidiolic acid ^a^	358.2144_16.34	C_22_H_30_O_4_	313.217/245.155/179.107	1	0.56	8.33	Sesquiterpene	Cannabinoid
43	Acequinocyl	384.2302_16.95	C_24_H_32_O_4_	193.050/165.055	4	0.25	4.17	Naphthoquinone derivative	Acaricide
44	2,2-Methylenebis(4-ethyl-6-tert-butylphenol)	368.2716_17.64	C_25_H_36_O_2_	191.143/135.080/163.112	3	0.23	4.17	Phenol antioxidant	Resin additive
45	Noscapine	413.1476_15.14	C_22_H_23_NO_7_	220.097/353.102/205.074	3	0.36	4.17	Isoquinoline alkaloid	Pharmaceutical
46	Deoxylimonin	454.1993_14.44	C_26_H_30_O_7_	165.070/178.078/202.078	3	0.39	4.17	Triterpene	NPC
47	Ursolic acid ^a^	456.3604_16.75	C_30_H_48_O_3_	119.086/133.101/107.086	1	0.03	20.83	Triterpene	NPC
48	Betulinic acid methyl ester	470.3762_19.02	C_31_H_50_O_3_	119.086/135.117/109.101	3	0.45	8.33	Triterpene	NPC
49	Limonin	470.1943_13.74	C_26_H_30_O_8_	161.060/141.070/133.065	3	0.49	4.17	Triterpene	NPC
50	Tau-fluvalinate ^a^	502.1274_16.70	C_26_H_22_ClF_3_N_2_O_3_	181.065/153.070/152.062	1	0.49	12	Pyrethroid	Insecticide
51	Kaempferitrin ^a^	578.1636_12.89	C_27_H_30_O_14_	287.055/121.028/153.018	1	0.01	4.17	Flavonoid glycoside	NPC

^a^: verified with injection of analytical standard solution, frequency % = percentage frequency of detection across the samples.

**Table 2:** Pesticide detections and concentrations (mg/kg) in honeybee samples using LC, GC-MS/MS.

		Active Substances Concentration ± SD (mg/kg) ^a^										
Sample Code	Location	Tau-Fluvalinate	Imidacloprid	Permethrin	Thiabendazole	Phenothrin	λ-Cyhalothrin	Bifenthrin	Carbaryl	Propiconazole	Fluopyram	Boscalid
A-23	Lefkada island	0.27 ± 0.08	n.d. *	n.d.	n.d.	n.d.	n.d.	n.d.	n.d.	0.0035	n.d.	<0.001
B-23		0.15 ± 0.04	n.d.	n.d.	n.d.	n.d.	n.d.	n.d.	n.d.	n.d.	n.d.	n.d.
C1-23	Lakonia	n.d.	0.0023 ± 0.0010	0.053 ± 0.010	n.d.	n.d.	n.d.	n.d.	<0.001 **	<0.001	n.d.	n.d.
C2-23		n.d.	n.d.	n.d.	n.d.	0.042	n.d.	n.d.	n.d.	n.d.	n.d.	n.d.
D-23	Chios island	n.d.	n.d.	n.d.	0.0018 ± 0.0010	0.95	n.d.	n.d.	n.d.	n.d.	n.d.	n.d.
E-23	Evros	0.89 ± 0.16	n.d.	n.d.	n.d.	n.d.	<0.001	n.d.	n.d.	n.d.	n.d.	n.d.
F-23	Evros	n.d.	n.d.	n.d.	n.d.	n.d.	n.d.	n.d.	n.d.	n.d.	<0.001	0.0054 ± 0.0014
G-23		n.d.	n.d.	n.d.	n.d.	n.d.	n.d.	n.d.	n.d.	n.d.	0.0011 ± 0.0005	n.d.
H-23	Crete	n.d.	n.d.	n.d.	n.d.	n.d.	n.d.	0.0073 ± 0.0021	n.d.	n.d.	n.d.	n.d.

^a^: n = 3, * n.d.: non-detected, ** 1-naphthol was tentatively annotated through LC-HRMS in this sample.

**Table 3:** Risk quotients, no-concern levels, and maximum concentrations of quantified pesticides.

Active Substance	RQoral	RQcontact	NCLoral (mg/kg)	NCLcontact (mg/kg)	Maximum Concentration (mg/kg)
Tau-fluvalinate	7.06 × 10^−3^	7.42 × 10^−3^	2.52	2.40	0.89
Imidacloprid	6.22 × 10^−2^	2.84 × 10^−3^	0.00074	0.0162	**0.0023** ^a^
Permethrin	4.08 × 10^−2^	2.21 × 10^−1^	0.026	0.0048	**0.053**
Phenothrin	5.94 × 10^−1^	7.31 × 10^−1^	0.032	0.026	**0.95**
λ-Cyhalothrin	3.19 × 10^−3^	7.63 × 10^−2^	0.182	0.0076	**0.29**
Bifenthrin	7.3 × 10^−3^	4.56 × 10^−2^	0.02	0.0032	**0.0073**
Thiabendazole	4.50 × 10^−5^	5.29 × 10^−6^	0.8	6.8	0.0018
Carbaryl	4.76 × 10^−4^	7.14 × 10^−4^	0.042	0.028	0.0010
Propiconazole	5.00 × 10^−6^	5.00 × 10^−6^	20	20	0.005
Fluopyram	1.08 × 10^−6^	1.10 × 10^−6^	20.46	20	0.0011
Boscalid	3.25 × 10^−6^	2.70 × 10^−6^	33.20	40	0.0054

^a^: in bold, concentrations that exceed one or both NCLs (oral and contact).

## Data Availability

The original contributions presented in this study are included in the article/Supplementary Materials. Further inquiries can be directed to the corresponding authors.

## Supplementary Materials

The following supporting information can be downloaded at: https://www.mdpi.com/article/10.3390/jox16020064/s1. Table S1: Method performance and validation for the newly validated active substances: Limits of Quantification (LOQ), ME (%), Recoveries (%), Repeatability (RSD%) and Inter-day (Inter-d) precision (RSD%) obtained in honey bees; Table S2: PFAS tentatively identified via in silico fragmentation using MetFrag Web tool in the honeybee samples; Figure S1: TIC MRM chromatogram of a standard mix solution of pesticides at 50 ng/mL in LC-ESI-MS/MS; Figure S2: Triisopropanolamine annotation based on MS/MS fragmentation pattern using mzCloud database. The top panel is the MS/MS pattern of the honey bee extract, whereas the bottom panel is the MS/MS pattern of Triisopropano-lamine from the mzCloud database; Figure S3: Acetophenone annotation based on MS/MS fragmentation pattern using mzCloud database. The top panel is the MS/MS pattern of the honey bee extract, whereas the bottom panel is the MS/MS pattern of Rimantadine from the mzCloud database; Figure S4: 1,5-Isoquinolinediol annotation based on MS/MS fragmentation pattern using mzCloud database. The top panel is the MS/MS pattern of the honey bee extract, whereas the bottom panel is the MS/MS pattern of 1,5-Isoquinolinediol from the mzCloud database; Figure S5: 1-Naphthol annotation based on MS/MS fragmentation pattern using mzCloud database. The top panel is the MS/MS pattern of the honey bee extract, whereas the bottom panel is the MS/MS pattern of 1-Naphthol from the mzCloud database; Figure S6: 2,2-Methylenebis(4-ethyl-6-tert-butylphenol) annotation based on MS/MS fragmentation pattern using mzCloud database. The top panel is the MS/MS pattern of the honey bee extract, whereas the bottom panel is the MS/MS pattern of 2,2-Methylenebis(4-ethyl-6-tert-butylphenol) from the mzCloud database; Figure S7: 2-Amino-6-methylmercaptopurine annotation based on MS/MS fragmentation pattern using mzCloud data-base. The top panel is the MS/MS pattern of the honey bee extract, whereas the bottom panel is the MS/MS pattern of 2-Amino-6-methylmercaptopurine from the mzCloud database; Figure S8: Arachidonic acid annotation based on MS/MS fragmentation pattern using mzCloud database. The top panel is the MS/MS pattern of the honey bee extract, whereas the bottom panel is the MS/MS pattern of Arachidonic acid from the mzCloud database; Figure S9: Betulinic acid methyl ester annotation based on MS/MS fragmentation pattern using mzCloud database. The top panel is the MS/MS pattern of the honey bee extract, whereas the bottom panel is the MS/MS pattern of Betulinic acid methyl ester from the NIST database; Figure S10: Deoxylimonin annotation based on MS/MS fragmentation pattern using mzCloud database. The top panel is the MS/MS pattern of the honey bee extract, whereas the bottom panel is the MS/MS pattern of Deoxylimonin from the mzCloud database; Figure S11: Diisobutylphthalate annotation based on MS/MS fragmentation pattern using mzCloud database. The top panel is the MS/MS pattern of the honey bee extract, whereas the bottom panel is the MS/MS pattern of Diisobu-tylphthalate from the mzCloud database; Figure S12: Dimethyl sebacate annotation based on MS/MS fragmentation pattern using mzCloud database. The top panel is the MS/MS pattern of the honey bee extract, whereas the bottom panel is the MS/MS pattern of Dimethyl seba-cate from the mzCloud database; Figure S13: D-Panthenol annotation based on MS/MS fragmentation pattern using mzCloud database. The top panel is the MS/MS pattern of the honey bee extract, whereas the bottom panel is the MS/MS pattern of D-Panthenol from the mzCloud database; Figure S14: Ethyl oleate annotation based on MS/MS fragmentation pattern using mzCloud database. The top panel is the MS/MS pattern of the honey bee extract, whereas the bottom panel is the MS/MS pattern of Ethyl oleate from the mzCloud database; Figure S15: Fenbendazole annotation based on MS/MS fragmentation pattern using mzCloud database. The top panel is the MS/MS pattern of the honey bee extract, whereas the bottom panel is the MS/MS pattern of Fenbendazole from the mzCloud database; Figure S16: Gabapentin annotation based on MS/MS fragmentation pattern using mzCloud database. The top panel is the MS/MS pattern of the honey bee extract, whereas the bottom panel is the MS/MS pattern of Gabapentin from the mzCloud database; Figure S17: Icaridin annotation based on MS/MS fragmentation pattern using mzCloud database. The top panel is the MS/MS pattern of the honey bee extract, whereas the bottom panel is the MS/MS pattern of Icaridin from the mzCloud database; Figure S18: Isobornyl methacrylate annotation based on MS/MS fragmentation pattern using mzCloud database. The top panel is the MS/MS pattern of the honey bee extract, whereas the bottom panel is the MS/MS pattern of Isobornyl methacrylate from the mzCloud database; Figure S19: Limonin annotation based on MS/MS fragmentation pattern using mzCloud database. The top panel is the MS/MS pattern of the honey bee extract, whereas the bottom panel is the MS/MS pattern of Limonin from the mzCloud database; Figure S20: N-Butylbenzenesulfonamide annotation based on MS/MS fragmentation pattern using mzCloud database. The top panel is the MS/MS pattern of the honey bee extract, whereas the bottom panel is the MS/MS pattern of N-Butylbenzenesulfonamide from the mzCloud database; Figure S21: Noscapine annotation based on MS/MS fragmentation pattern using mzCloud database. The top panel is the MS/MS pattern of the honey bee extract, whereas the bottom panel is the MS/MS pattern of Noscapine from the mzCloud database; Figure S22: Scopolamine annotation based on MS/MS fragmentation pattern using mzCloud database. The top panel is the MS/MS pattern of the honey bee extract, whereas the bottom panel is the MS/MS pattern of Scopolamine from the mzCloud database; Figure S23: Skatole annotation based on MS/MS fragmentation pattern using mzCloud database. The top panel is the MS/MS pattern of the honey bee extract, whereas the bottom panel is the MS/MS pattern of Skatole from the mzCloud database; Figure S24: Stylopine annotation based on MS/MS fragmentation pattern using mzCloud database. The top panel is the MS/MS pattern of the honey bee extract, whereas the bottom panel is the MS/MS pattern of Stylopine from the NIST da-tabase; Figure S25: Syringate annotation based on MS/MS fragmentation pattern using mzCloud database. The top panel is the MS/MS pattern of the honey bee extract, whereas the bottom panel is the MS/MS pattern of Syringate from the NIST da-tabase; Figure S26: Trans-Cinnamaldehyde annotation based on MS/MS fragmentation pattern using mzCloud database. The top panel is the MS/MS pattern of the honey bee extract, whereas the bottom panel is the MS/MS pattern of trans-Cinnamaldehyde from the mzCloud database; Figure S27: MRM chromatograms of phenothrin detection in bee sample (108-23); Figure S28: Potential chemical structures for PFAS compounds 2 and 14; Excel file: MRM EXCEL_Bees_paper. Reference [80] is cited in the Supplementary Materials.

## Abbreviations

The following abbreviations are used in this manuscript:

LC-HRMS	liquid chromatography-high-resolution mass spectrometry
HRMS	High-Resolution Mass Spectrometry
LC, GC-MS/MS	liquid and gas chromatographic tandem mass spectrometry
PFAS	Per- and Polyfluoroalkyl Substances
LOQ	limit of quantification
NTS	non-targeted screening
