# Supplementary material for: Non-Targeted and Targeted Screening of Organic Contaminants in Honeybees’ Death Incidents in Greece: A Story Beyond Pesticides
Source: J Xenobiot. 2026 Apr 8;16(2):64. doi: 10.3390/jox16020064 (PMC13117638; doi:10.3390/jox16020064)
Supplement: Supplementary file 1 [file jox-16-00064-s001.zip › jox-4216849-supplementary.pdf]

Journal of Xenobiotics

Supplementary materials: Non-Targeted and Targeted Screening of Organic Contaminants in Honeybees' Death Incidents in Greece: A Story Beyond Pesticides

Eirini Baira, Evangelia N. Tzanetou, Electra Manea-Karga, Kyriaki Machera and Konstantinos M. Kasiotis

Contents

Sample preparation

Analytical Method Validation

Table S1. Method performance and validation for the newly validated active substances: Limits of Quantification (LOQ), ME (%), Recoveries (%), Repeatability (RSD%) and Inter-day (Inter-d) precision (RSD%) obtained in honey bees

Table S2. PFAS tentatively identified via *in silico* fragmentation using MetFrag Web tool in the honeybee samples

Figure S1. TIC MRM chromatogram of a standard mix solution of pesticides at 50 ng/mL in LC-ESI-MS/MS

Figure S2. Triisopropanolamine annotation based on MS/MS fragmentation pattern using mzCloud database. The top panel is the MS/MS pattern of the honey bee extract, whereas the bottom panel is the MS/MS pattern of Triisopropanolamine from the mzCloud database.

Figure S3. Acetophenone annotation based on MS/MS fragmentation pattern using mzCloud database. The top panel is the MS/MS pattern of the honey bee extract, whereas the bottom panel is the MS/MS pattern of Rimantadine from the mzCloud database.

Figure S4. 1,5-Isoquinolinediol annotation based on MS/MS fragmentation pattern using mzCloud database. The top panel is the MS/MS pattern of the honey bee extract, whereas the bottom panel is the MS/MS pattern of 1,5-Isoquinolinediol from the mzCloud database.

Figure S5. 1-Naphthol annotation based on MS/MS fragmentation pattern using mzCloud database. The top panel is the MS/MS pattern of the honey bee extract, whereas the bottom panel is the MS/MS pattern of 1-Naphthol from the mzCloud database.

Figure S6. 2,2-Methylenebis(4-ethyl-6-tert-butylphenol) annotation based on MS/MS fragmentation pattern using mzCloud database. The top panel is the MS/MS pattern of the honey bee extract, whereas the bottom panel is the MS/MS pattern of 2,2-Methylenebis(4-ethyl-6-tert-butylphenol) from the mzCloud database.

Figure S7. 2-Amino-6-methylmercaptopurine annotation based on MS/MS fragmentation pattern using mzCloud database. The top panel is the MS/MS pattern of the honey bee extract, whereas the bottom panel is the MS/MS pattern of 2-Amino-6-methylmercaptopurine from the mzCloud database.

Figure S8. Arachidonic acid annotation based on MS/MS fragmentation pattern using mzCloud database. The top panel is the MS/MS pattern of the honey bee extract, whereas the bottom panel is the MS/MS pattern of Arachidonic acid from the mzCloud database.

Figure S9. Betulinic acid methyl ester annotation based on MS/MS fragmentation pattern using mzCloud database. The top panel is the MS/MS pattern of the honey bee extract, whereas the bottom panel is the MS/MS pattern of Betulinic acid methyl ester from the NIST database.

Figure S10. Deoxylimonin annotation based on MS/MS fragmentation pattern using mzCloud database. The top panel is the MS/MS pattern of the honey bee extract, whereas the bottom panel is the MS/MS pattern of Deoxylimonin from the mzCloud database.

Figure S11. Diisobutylphthalate annotation based on MS/MS fragmentation pattern using mzCloud database. The top panel is the MS/MS pattern of the honey bee extract, whereas the bottom panel is the MS/MS pattern of Diisobutylphthalate from the mzCloud database.

Figure S12. Dimethyl sebacate annotation based on MS/MS fragmentation pattern using mzCloud database. The top panel is the MS/MS pattern of the honey bee extract, whereas the bottom panel is the MS/MS pattern of Dimethyl sebacate from the mzCloud database.

Figure S13. D-Panthenol annotation based on MS/MS fragmentation pattern using mzCloud database. The top panel is the MS/MS pattern of the honey bee extract, whereas the bottom panel is the MS/MS pattern of D-Panthenol from the mzCloud database.

Figure S14. Ethyl oleate annotation based on MS/MS fragmentation pattern using mzCloud database. The top panel is the MS/MS pattern of the honey bee extract, whereas the bottom panel is the MS/MS pattern of Ethyl oleate from the mzCloud database.

Figure S15. Fenbendazole annotation based on MS/MS fragmentation pattern using mzCloud database. The top panel is the MS/MS pattern of the honey bee extract, whereas the bottom panel is the MS/MS pattern of Fenbendazole from the mzCloud database.

Figure S16. Gabapentin annotation based on MS/MS fragmentation pattern using mzCloud database. The top panel is the MS/MS pattern of the honey bee extract, whereas the bottom panel is the MS/MS pattern of Gabapentin from the mzCloud database.

Figure S17. Icaridin annotation based on MS/MS fragmentation pattern using mzCloud database. The top panel is the MS/MS pattern of the honey bee extract, whereas the bottom panel is the MS/MS pattern of Icaridin from the mzCloud database.

Figure S18. Isobornyl methacrylate annotation based on MS/MS fragmentation pattern using mzCloud database. The top panel is the MS/MS pattern of the honey bee extract, whereas the bottom panel is the MS/MS pattern of Isobornyl methacrylate from the mzCloud database.

Figure S19. Limonin annotation based on MS/MS fragmentation pattern using mzCloud database. The top panel is the MS/MS pattern of the honey bee extract, whereas the bottom panel is the MS/MS pattern of Limonin from the mzCloud database.

Figure S20. N-Butylbenzenesulfonamide annotation based on MS/MS fragmentation pattern using mzCloud database. The top panel is the MS/MS pattern of the honey bee extract, whereas the bottom panel is the MS/MS pattern of N-Butylbenzenesulfonamide from the mzCloud database.

Figure S21. Noscapine annotation based on MS/MS fragmentation pattern using mzCloud database. The top panel is the MS/MS pattern of the honey bee extract, whereas the bottom panel is the MS/MS pattern of Noscapine from the mzCloud database.

Figure S22. Scopolamine annotation based on MS/MS fragmentation pattern using mzCloud database. The top panel is the MS/MS pattern of the honey bee extract, whereas the bottom panel is the MS/MS pattern of Scopolamine from the mzCloud database.

Figure S23. Skatole annotation based on MS/MS fragmentation pattern using mzCloud database. The top panel is the MS/MS pattern of the honey bee extract, whereas the bottom panel is the MS/MS pattern of Skatole from the mzCloud database.

Figure S24. Stylopine annotation based on MS/MS fragmentation pattern using mzCloud database. The top panel is the MS/MS pattern of the honey bee extract, whereas the bottom panel is the MS/MS pattern of Stylopine from the NIST database.

Figure S25. Syringate annotation based on MS/MS fragmentation pattern using mzCloud database. The top panel is the MS/MS pattern of the honey bee extract, whereas the bottom panel is the MS/MS pattern of Syringate from the NIST database.

Figure S26. Trans-Cinnamaldehyde annotation based on MS/MS fragmentation pattern using mzCloud database. The top panel is the MS/MS pattern of the honey bee extract, whereas the bottom panel is the MS/MS pattern of trans-Cinnamaldehyde from the mzCloud database.

Figure S27. MRM chromatograms of phenothrin detection in bee sample

Figure S28. Potential chemical structures of PFAS compounds 2 and 14

Sample preparation

Specifically, 1 g of bees was fortified at appropriate concentrations with the Internal Standards and then homogenized with 3 mL of deionized water and 7 mL of acetonitrile. Then the mixture was vortex-mixed with 0.5 g of magnesium

sulfate anhydrous ($MgSO_4$) and 0.2 g of sodium acetate (NaOAc). The samples were then centrifuged for 5 minutes at 4500 rpm. Approximately 6 mL of acetonitrile was transferred to a 15 mL Falcon tube containing the clean-up salts (PSA and $MgSO_4$). The tube was vigorously shaken for 1 min and then centrifuged (same conditions as above). Then, the final extract was divided into two equal parts: The first aliquot was evaporated under a gentle stream of nitrogen and then reconstituted with 1 mL of a 75:25 (v/v) MeOH:H_2O solution for LC-MS/MS analysis. The second aliquot after evaporation (for GC-MS/MS analysis) was reconstituted using acetonitrile prior to injection into the chromatographic system.

Analytical Method Validation

For linearity, the concentration range varied from 0.5 to 500 ng/mL assessed at seven concentration levels (for a TIC MRM chromatogram see Figure 1). Apart from correlation coefficients ($r^2 \geq 0.99$), residuals from regression analysis were evaluated, and should be below ±20%. Three individually prepared replicates at each concentration were analyzed. The results (mean, standard deviation, relative standard deviation-RSD) were recorded and were evaluated as acceptable. LOQ was defined as the lowest spike level meeting the method performance criteria for trueness and precision. Trueness was assessed *via* recovery study. Therefore, spiked samples were prepared at three concentrations using five replicates (n=5) per level (LOQ, 10 × LOQ, 100 × LOQ). Acceptable mean recoveries should fluctuate between 70-120% (or in routine analysis within the realistic default range of 60-140%) as outlined in the SANTE guideline. Precision was articulated as RSD% of the intra-day (RSDr) and within-laboratory reproducibility (RSDwR), analyses (n=5) over 1, 2, and 3 days, respectively, for the spike levels tested. Specificity was assessed through replicate injections of blank control and reagent blank samples. Matrix Effect (ME) was assessed at the LOQ and at 10 × LOQ with six replicates per level, by comparing the response of solvent standards and matrix-matched standards (Matuszewski et al., 2003).
Analytical method validation proved the efficiency of the LC,GC-MS/MS methods. More specifically, recoveries were tested at three concentration levels (LOQ, 10×LOQ, and 100×LOQ) and were found in the range of 63-119% (with acceptable %RSD values, see Table S1 below) for all the target compounds (analyzed in LC-MS/MS and GC-MS/MS). Matrix effects % (see Table S1 for ME% at the LOQ, complete data not shown for 10×LOQ) verified slight ME enhancement or suppression. The residuals from the regression analysis did not exceed ±16%. The uncertainty investigation showed that the expanded measurement uncertainty varied at LOQ from 16.04 to 48.39%, and at 10×LOQ from 12.16-42.81%. Lastly, the retention times of the analytes fell within the threshold criterion of ±0.1 min.

Table S1. Method performance and validation for the newly validated active substances: Limits of Quantification (LOQ), ME (%), Recoveries (%), Repeatability (RSD%) and Inter-day (Inter-d) precision (RSD%) obtained in honey bees.[a]

**Analytical Method**										
				**Recovery ±RSD % (intra-day precision),** n = 3			**Recovery ±RSD % (inter-day precision),** n = 3			**MRM ratio (at LOQ)**
	**Analyte**	**LOQ (ng/g)**	**ME (%)**	LOQ	10×LOQ	100×LOQ	LOQ	10×LOQ	100×LOQ	
LC-ESI-QqQ-MS	Acephate	1	110	75 ± 10	82 ± 13	85 ± 14	74 ± 11	85 ± 11	91 ± 10	20
	Acetamiprid	0.5	99	93 ± 7	97 ± 10	101 ± 12	94 ± 7	88 ± 10	93 ± 5	18
	Aldicarb	0.5	109	95 ± 11	87 ± 8	92 ± 10	85 ± 8	81 ± 12	85 ± 11	72
	Aldicarb sulfone	0.5	124	94 ± 15	103 ± 10	112 ± 11	105 ± 11	103 ± 9	97 ± 11	16
	Aldicarb sulfoxide	0.5	121	102 ± 16	114 ± 11	108± 10	96 ± 9	97 ± 11	92 ± 11	82
	Ametoctradin	5	118	69 ± 12	82 ± 11	77 ± 17	70 ± 15	81 ± 14	80 ± 14	24
	Amisulbrom	5	119	86 ± 11	81 ± 13	84 ± 12	92 ± 16	80 ± 15	80 ± 11	33
	Amitraz	5	93	103 ± 15	110 ± 11	112 ± 14	107 ± 5	116 ± 4	114 ± 14	45
	Atrazine-2-hydroxy	5	88	72 ± 14	73 ± 16	81 ± 12	71 ± 15	82 ± 2	87 ± 3	15
	Avermectin B1a	5	91	78 ± 6	81 ± 8	83 ± 10	80 ± 5	79 ± 9	97 ± 6	39
	Avermectin B1b	5	90	80 ± 11	81 ± 14	87 ± 13	79 ±12	80 ± 15	84 ± 16	42
	Azinphos methyl	5	107	79 ± 9	83 ± 11	84 ± 9	90 ± 8	89 ± 10	92 ± 7	72
	Azoxystrobin	1	118	74 ± 11	81 ± 13	97 ± 13	100 ± 14	102 ± 11	97 ± 12	44
	Bitertanol	5	124	77 ± 14	72 ± 10	76 ± 12	73 ± 10	96 ± 8	97 ± 12	68
	Boscalid	1	117	74 ± 14	81 ± 15	97 ± 15	88 ± 4	93 ± 5	97 ± 7	36
	Bromacil	5	131	84 ± 9	73 ± 12	78 ± 11	83 ± 13	76 ± 16	94 ± 13	15
	Bromuconazole	1	95	88 ± 10	91 ± 12	95 ± 12	90 ± 12	93 ± 14	98 ± 6	32
	Butocarboxim	1	122	85 ± 8	92 ± 10	104 ± 13	96 ± 7	97 ± 11	96 ± 10	39
	Carbaryl	1	98	81 ± 6	88 ± 7	95 ± 14	80 ± 14	84 ± 7	91 ± 14	78
	Carbendazim	0.5	119	94 ± 9	106 ± 11	111 ± 12	88 ± 8	89 ± 10	96 ± 12	26
	Carbetamide	1	110	79 ± 6	81 ± 9	87 ± 10	106 ± 3	111 ±5	117 ± 5	10
	Carbofuran	1	88	92 ± 10	83 ± 11	97 ± 11	89 ± 15	83 ±16	91 ± 15	56

https://doi.org/10.3390/xxxxx

LC-ESI-QqQ-MS	Carbofuran 3 hydroxy	5	86	70 ± 15	76 ± 11	84 ± 12	89 ± 10	96 ± 11	98 ± 12	67
	Carbosulfan	5	92	85 ± 11	87 ± 12	94 ± 11	85 ± 12	88 ± 10	91 ± 14	30
	Chlorantraniliprole	1	112	86 ± 7	81 ± 9	86 ± 13	81 ± 9	84 ± 13	79 ± 12	93
	Chloridazon	1	126	78 ± 11	74 ± 11	82 ± 12	81 ± 16	76 ± 17	108 ±17	57
	Chlorobromuron	1	85	78 ± 10	84 ± 10	87 ± 12	80 ± 7	87 ± 8	93 ± 9	80
	Chloroxuron	1	120	95 ± 9	98 ± 10	102 ± 10	85 ± 9	88 ± 14	95 ± 9	48
	Chlorpropham	1	107	89 ± 8	78 ± 10	81 ± 10	86 ± 10	80 ± 11	78 ± 11	71
	Chlorpyriphos ethyl	1	114	79 ± 7	87 ± 13	80 ± 11	77 ± 12	83 ± 12	77 ± 13	25
	Chlorpyrifos oxon	1	113	84 ± 11	85 ± 11	90 ± 7	81 ± 13	77 ± 10	83 ± 9	36
	Chlorpyriphos methyl	1	98	81 ± 15	80 ± 14	78 ± 12	79 ± 13	74 ± 13	79 ± 10	40
	Clopyralid	1	87	83 ± 10	78 ± 12	82 ± 12	80 ± 11	91 ± 12	93 ± 12	51
	Coumaphos	1	92	86 ± 9	96 ± 12	116 ± 6	99 ± 12	101 ± 12	103 ± 11	39
	Coumaphos oxon	1	110	94 ± 12	104 ± 9	112 ± 12	75 ± 13	83 ± 12	85 ± 12	57
	Demeton S methyl sulfone	1	110	89 ± 7	76 ± 11	85 ± 13	77 ± 14	79 ± 13	80 ±17	45
	Dichlofluanid	5	98	74 ± 7	87 ± 8	85 ± 5	76 ± 9	89 ± 10	81 ± 11	77
	Dichlorvos	1	83	81 ± 8	76 ± 11	83 ± 11	75 ± 8	73 ± 10	82 ± 14	23
	Dicrotofos (dicrotophos)	5	118	84 ± 10	88 ± 11	89 ± 12	88 ± 10	77 ± 16	100 ± 11	29
	Diflubenzuron	1	122	79 ± 11	85 ± 9	91 ± 11	81 ± 10	76 ± 10	84 ± 13	75
	Dimethoate	1	115	79 ± 10	95 ± 10	108 ± 11	103 ± 4	105 ± 5	107 ± 5	49
	Dimethomorph	1	88	93 ± 9	104 ± 13	112 ± 12	99 ± 5	101 ± 3	103 ± 3	58
	Diniconazole	5	127	85 ± 7	91 ± 8	98 ± 8	80 ± 11	85 ± 10	93 ± 10	75
	Diphenylamine	1	110	77 ± 11	95 ± 11	109 ± 10	97 ± 10	99 ± 11	102 ± 11	21
	Diuron	1	85	81 ± 10	98 ± 10	92 ± 11	91 ± 5	97 ± 5	99 ± 5	65
	DMF	1	79	108 ± 13	114 ± 14	118 ± 11	113 ± 10	116 ± 11	119 ± 11	18
	DMPF	1	85	110 ± 11	114 ± 13	119 ± 13	104 ± 5	107 ± 6	109 ± 8	60

	Emamectin B1a	5	88	64 ± 15	69 ± 14	73 ± 14	76 ± 8	86 ± 10	88 ± 11	30
	Emamectin B1b	5	89	69 ± 17	71 ± 16	76 ± 16	79 ± 13	86 ± 11	92 ± 10	27
LC-ESI-QqQ-MS	Epoxiconazole	1	106	79 ± 11	88 ± 14	93 ± 13	74 ± 12	87 ± 15	90 ± 12	41
	Ethirimol	5	118	78 ± 9	81 ± 9	93 ± 11	81 ± 90	78 ± 12	85 ± 14	87
	Ethofumesate	1	120	72 ± 11	79 ± 10	83 ± 11	74 ± 13	79 ± 10	83 ± 11	60
	Ethoxyquin	1	122	71 ± 7	74 ± 9	80 ± 9	70 ± 10	75 ± 9	77 ± 12	39
	Etofenprox	1	87	90 ± 11	93 ± 9	105 ± 10	91 ± 9	87 ± 11	96 ± 15	78
	Etoxazole	5	114	78 ± 6	82 ± 7	89 ± 8	76 ± 9	81 ± 10	85 ± 12	73
	Fenamiphos	5	115	73 ± 11	74 ± 8	80 ± 11	72 ± 8	80 ± 12	83 ± 13	54
	Fenamiphos sulfone	1	91	76 ± 10	89 ± 11	95 ± 12	72 ± 11	84 ± 14	90 ± 16	40
	Fenamiphos sulfoxide	1	9	78 ± 11	72 ± 10	87 ± 13	75 ± 12	70 ± 10	83 ± 15	66
	Fenarimol	1	102	73 ± 9	77 ± 9	84 ± 10	69 ± 9	83 ± 8	84 ± 14	50
	Fenazaquin	5	98	65 ± 12	81 ± 13	84 ± 13	68 ± 13	75 ± 15	81 ± 12	39
	Fenhexamide	1	94	71 ± 11	77 ± 10	82 ± 10	68 ± 10	75 ± 12	83 ± 11	65
	Fenitrothion	1	80	86 ± 7	91 ± 5	97 ± 9	82 ± 9	92 ± 5	90 ± 12	50
	Fenoxycarb	1	93	80 ± 10	82 ± 9	88 ± 10	76 ± 12	84 ± 12	87 ± 14	33
	Fenpropidin	1	103	78 ± 10	83 ± 8	96 ± 6	81 ± 13	77 ± 14	87 ± 10	53
	Fenpropymorph	5	103	68 ± 8	74 ± 7	84 ± 7	69 ± 7	79 ± 10	84 ± 13	50
	Fenpyroximate	1	127	72 ± 11	75 ± 11	86 ± 10	68 ± 11	79 ± 14	80 ± 10	8
	Fenthion oxon (fenoxon)	1	87	84 ± 10	89 ± 10	92 ± 11	81 ± 9	89 ± 10	92 ± 11	43
	Fenthion oxon sulfone	1	89	81 ± 7	92 ± 7	96 ± 9	75 ± 12	90 ± 9	89 ± 16	38
	Fenthion oxon sullfoxide	1	98	64 ± 14	68 ± 13	81 ± 11	67 ± 13	73 ± 13	76 ± 10	65
	Fenthion sulfone	1	117	73 ± 8	81 ± 9	97 ± 11	75 ± 10	78 ± 11	86 ± 16	74
	Fluazifop	5	89	76 ± 11	84 ± 10	89 ± 10	68 ± 12	80 ± 15	85 ± 13	46
	Fluazifop-P-butyl	5	91	89 ± 7	91 ± 6	94 ± 7	83 ± 12	83 ± 9	90 ± 11	59
	Flufenoxuron	1	119	72 ± 8	76 ± 9	84 ± 9	75 ± 10	82 ± 8	88 ± 15	93
	Fluometuron	5	130	78 ± 11	89 ± 10	92 ± 12	71 ± 11	80 ± 12	81 ± 10	59

	Fluopicolide	1	105	64 ± 12	75 ± 16	84 ± 14	67 ± 13	75 ± 15	79 ± 15	27
	Fluopyram	1	103	77 ± 12	82 ± 13	87 ± 13	74 ± 11	80 ± 15	82 ± 10	58
LC-ESI-QqQ-MS	Fluquin-conazole	1	121	76 ± 13	84 ± 14	92 ± 11	73 ± 11	80 ± 13	90 ± 16	29
	Flutriafol	1	89	83 ± 11	83 ± 10	80 ± 10	76± 10	81 ± 14	80 ± 12	33
	Fluxapyroxad	1	90	68 ± 15	71 ± 14	84 ± 14	71 ± 15	76 ± 12	81 ± 15	19
	Furathiocarb	1	108	78 ± 10	75 ± 14	77 ± 8	81 ± 15	74 ± 11	77 ± 12	51
	Haloxyfop	5	119	72 ± 10	78 ± 10	83 ± 8	70 ± 9	72 ± 15	81 ± 9	60
	Heptenophos	5	121	80 ± 14	74 ± 14	77 ± 13	82 ± 14	73 ± 17	80 ± 15	71
	Hexaconazole	1	99	81 ± 7	83 ± 6	85 ± 7	74 ± 9	72 ± 13	70 ± 8	50
	Hexythiazox	5	101	67 ± 11	73 ± 12	82 ± 12	69 ± 13	74 ± 15	77 ± 12	22
	Imidacloprid	0.5	110	84 ± 11	94 ± 11	92 ± 10	80 ± 13	90 ± 11	79 ± 15	44
	Isoprothiolane	5	98	79 ± 7	77 ± 9	74 ± 9	81 ± 10	75 ± 13	73 ± 11	48
	Malathion	5	120	101 ± 11	114 ± 10	119 ± 10	89 ± 14	94 ± 10	90 ± 14	91
	Mepanipyrim	5	125	81 ± 7	87 ± 8	94 ± 8	73 ± 10	77 ± 14	84 ± 14	81
	Metamitron	1	90	78 ± 10	77 ± 9	76 ± 9	80 ± 10	76 ± 12	82 ± 9	69
	Metazachlor	1	113	75 ± 14	79 ± 12	83 ± 13	78 ± 8	85 ± 15	81 ± 16	70
	Methamidophos	5	115	83 ± 12	89 ± 12	86 ± 11	82 ± 16	89 ± 12	86 ± 11	36
	Methiocarb	1	131	66 ± 12	72 ± 13	76 ± 15	68 ± 17	75 ± 14	77 ± 17	13
	Methiocarb sul-fone	1	117	73 ± 12	84 ± 12	89 ± 11	70 ± 12	78 ± 11	85 ± 14	39
	Methiocarb sul-foxide	1	118	69 ± 8	71 ± 9	76 ± 8	70 ± 12	74 ± 13	74 ± 12	65
	Methomyl	1	98	84 ± 11	92 ± 11	94 ± 13	75 ± 13	84 ± 15	89 ± 14	21
	Metobromuron	1	119	77 ± 13	74 ± 14	72 ± 13	78 ± 14	70 ± 13	76 ± 10	81
	Metolachlor	1	124	89 ± 11	93 ± 9	101 ± 11	86± 11	90 ± 12	104 ± 15	49
	Metraferone	5	117	97 ± 10	103 ± 10	116 ± 9	90 ± 12	98 ± 15	106 ± 10	40
	Mevinphos cis/trans	1	127	74 ± 12	84 ± 14	97 ± 12	74 ± 12	84 ± 14	97 ± 12	54
	Myclobutanil	1	88	79 ± 11	87 ± 12	96 ± 13	81 ± 14	85 ± 16	90 ± 14	21
	Napropamide	1	81	68 ± 7	73 ± 9	80 ± 7	66 ± 9	73 ± 9	80 ± 7	58
	Nitenpyram	1	99	76 ± 11	79 ± 8	84 ± 10	78 ± 14	75 ± 8	80 ± 14	63
	Omethoate	1	79	80 ± 12	81 ± 11	87 ± 11	76 ± 15	77 ± 13	82 ± 14	52
	Oxadixyl	1	77	70 ± 9	78 ± 7	81 ± 10	71 ± 12	75 ± 10	74 ± 11	75

	Oxamyl	1	100	81 ± 12	80 ± 13	78 ± 10	76 ± 14	83 ± 13	75 ± 14	52
LC-ESI-QqQ-MS	Oxyfluorfen	5	109	72 ± 10	78 ± 11	84 ± 11	71 ± 13	76 ± 10	81 ± 16	47
	Paclobutazole	5	123	78 ± 11	85 ± 12	91 ± 12	76 ± 10	82 ± 14	87 ± 9	30
	Parathion ethyl	5	121	75 ± 7	74 ± 9	83 ± 9	70 ± 7	75 ± 11	80 ± 11	41
	Parathion methyl	5	114	78 ± 14	69 ± 13	75 ± 13	81 ± 14	70 ± 15	74 ± 12	30
	Pencycuron	5	128	67 ± 12	75 ± 13	81 ± 13	65 ± 11	77 ± 14	75 ± 15	9
	Pendimethalin	5	130	81 ± 9	79 ± 11	85 ± 9	80 ± 12	84 ± 15	82 ± 10	18
	Phosmet oxon	5	98	80 ± 15	84 ± 14	79 ± 14	82 ± 14	81 ± 14	76 ± 15	27
	Phoxim	1	90	66 ± 12	72 ± 11	84 ± 11	65 ± 14	74 ± 14	82 ± 11	18
	Picoxystrobin	1	80	74 ± 13	86 ± 12	91 ± 12	70 ± 12	82 ± 8	85 ± 13	16
	Pirimicarb desmethyl	1	110	80 ± 14	74 ± 13	79 ± 14	81 ± 15	72 ± 10	78 ± 12	38
	Procloraz	1	94	72 ± 11	77 ± 10	81 ± 10	74 ± 11	75 ± 12	77 ± 8	69
	Propamocarb	1	98	74 ± 9	85 ± 10	90 ± 9	70 ± 7	82 ± 8	90 ± 17	29
	Propargite	1	119	69 ± 11	79 ± 10	83 ± 11	64 ± 12	75 ± 10	79 ± 12	62
	Prothiofos	1	98	74 ± 13	68 ± 14	70 ± 13	72 ± 15	69 ± 14	67 ± 15	33
	Pymethrozine	5	132	76 ± 11	84 ± 9	89 ± 10	71 ± 11	80 ± 13	82 ± 10	72
	Pyridaben	1	93	83 ± 9	81 ± 10	79 ± 10	80 ± 9	74 ± 13	76 ± 12	71
	Pyrifenox	1	79	81 ± 7	84 ± 8	82 ± 8	75 ± 10	81 ± 13	77 ± 14	55
	Pyriproxyfen	0.5	98	82 ± 6	80 ± 7	85 ± 7	78 ± 9	82 ± 12	80 ± 13	25
	Quinalphos	1	108	65 ± 15	73 ± 16	78 ± 15	63 ± 12	71 ± 16	74 ± 17	50
	Quinoxyfen	1	126	64 ± 12	77 ± 11	83 ± 11	65 ± 11	67 ± 11	80 ± 10	40
	Quizalofop	1	121	66 ± 13	79 ± 12	84 ± 12	65 ± 15	80± 14	80 ± 12	37
	Spinosad (D)	1	88	66 ± 11	74 ± 10	89 ± 11	70 ± 14	75 ± 11	85 ± 12	14
	Spirodiclofen	1	98	72 ± 9	78 ± 11	92 ± 11	65 ± 11	80 ± 14	89 ± 10	28
	Sulfoxaflor	1	105	74 ± 11	82 ± 10	90 ± 11	75 ± 12	84 ± 12	92 ± 11	32
	Tebuconazole	1	120	86 ± 14	91 ± 13	99 ± 14	75 ± 14	90 ± 13	95 ± 14	23
	Tebufenpyrad	1	134	79 ± 6	88 ± 9	92 ± 9	75 ± 8	90 ± 9	91 ± 13	48
	Temephos	1	117	73 ± 11	80 ± 11	89 ± 10	75 ± 13	78 ± 13	90 ± 10	82
	Terbutryn	1	79	78 ± 6	82 ± 7	87 ± 6	70 ± 8	79 ± 9	83 ± 8	19
	Tetrachlorvinphos	1	101	84 ± 12	89 ± 11	94 ± 11	85 ± 10	87 ± 11	90 ± 11	38
	Tetraconazole	5	87	69 ± 14	74 ± 13	84 ± 13	69 ± 11	72 ± 13	89 ± 11	60

https://doi.org/10.3390/xxxxx

LC-ESI-QqQ-MS	Thiobencarb	1	100	73 ± 11	82 ± 10	93 ± 11	74 ± 12	80 ± 10	96 ± 10	27
	Thiophanate methyl	1	112	68 ± 12	74 ± 13	79 ± 13	70 ± 11	80 ± 14	81 ± 13	24
	Triadimefon	1	116	83 ± 11	83 ± 10	91 ± 10	80 ± 10	79 ± 10	87 ± 14	38
	Triadimenol	1	127	79 ± 14	88 ± 15	91 ± 15	72 ± 12	85 ± 14	90 ± 14	44
	Trifloxystrobin	5	114	77 ± 19	82 ± 17	88 ± 16	80 ± 17	80 ± 16	90 ± 13	59
	Triflumizole	5	112	70 ± 14	74 ± 13	86 ± 14	71 ± 13	76 ± 13	88± 13	49
				**Recovery ±RSD % (intra-day precision),** n = 3			**Recovery ±RSD % (inter-day precision),** n = 3			
	**Analyte**	**LOQ (ng/g)**		LOQ	10LOQ	100 LOQ	LOQ	10LOQ	100LOQ	
GC-EI-QqQ-MS	Acrinathrin	1	115	105 ± 12	103 ± 13	95 ± 14	98 ± 11	98 ± 14	92 ± 12	27
	Aldrin	1	95	95 ± 11	100 ± 12	105 ± 11	96 ± 11	102 ± 11	105 ± 10	30
	Azinphos ethyl	5	113	75 ± 16	81 ± 8	98 ± 9	73 ± 17	82 ± 8	100 ± 10	94
	Bifenthrin	1	121	95 ± 9	94 ± 10	92 ± 10	92 ± 10	92 ± 9	96 ± 11	20
	Boscalid	1	110	90 ± 8	96 ± 6	100 ± 6	93 ± 8	98 ± 7	102 ± 6	51
	Bromopropylate	1	127	94 ± 11	95 ± 13	98 ± 13	95 ± 12	95 ± 11	99 ± 11	73
	Chlordane a	1	99	94 ± 10	100 ± 11	105 ± 11	95 ± 12	101 ± 15	105 ± 12	79
	Chlorfenvinfos	1	119	76 ± 10	82 ± 10	99 ± 8	80 ± 15	83 ± 12	99 ± 12	84
	Chlorobeniz-ilate	5	130	85 ± 10	90 ± 9	102 ± 7	86 ± 10	89 ± 10	105 ± 9	32
	Chlorthal- di-methyl	1	132	110 ± 7	105 ± 6	105 ± 7	105 ± 9	102 ± 11	99 ± 10	94
	Cyfluthrin	1	106	85 ± 10	90 ± 9	102 ± 5	90 ± 12	99 ± 11	108 ± 8	82
	λ-Cyhalothrin	1	109	80 ± 14	85 ± 13	108 ± 10	86 ± 15	82 ± 12	101 ± 11	48
	Cypermethrin	1	99	85 ± 15	95 ± 12	100 ± 10	86 ± 14	90± 11	105 ± 11	93
	DDT (p,p′)	1	100	110 ± 7	105 ± 6	104 ± 6	106 ± 14	101± 10	105 ± 11	53
	DDE (p,p′)	1	103	95 ± 8	100 ± 8	102 ± 7	100 ± 13	101± 10	105 ± 11	70
	DDD (p,p′)	1	97	92± 9	95 ± 9	100 ± 8	95 ± 11	98 ± 10	99 ± 11	73
	Deltamethrin	1	111	77 ± 9	82 ± 5	98 ± 5	76 ± 12	85 ±81	99 ± 7	62
	Dicofol op	1	93	92 ± 11	95 ± 8	105 ± 7	89 ± 14	99 ± 11	110 ± 11	16
	Dicofol (p,p′)	1	92	88 ± 9	99 ± 5	105 ± 6	85 ± 13	100 ± 8	102 ± 9	21
GC-EI-QqQ-MS	Dieldrin	1	102	88 ± 7	99 ± 6	105 ± 4	90 ± 12	95 ± 9	99 ± 5	81
	Endosulfan a	1	117	63 ± 10	67 ± 9	72 ± 9	65 ± 11	69 ± 10	71 ± 11	30

https://doi.org/10.3390/xxxxx

Endosulfan b	1	113	65 ± 10	71 ± 11	76 ± 9	63 ± 13	70 ± 11	73 ± 9	28
Endosulfan sulfate	1	88	65 ± 10	69 ± 12	74 ± 11	63 ± 15	70 ± 11	75 ± 10	19
Endrin	1	92	84 ± 6	87 ± 4	99 ± 5	87 ± 8	95 ± 7	99 ± 7	83
Esfenvalerate	1	82	82 ± 8	87 ± 7	95 ± 7	87 ± 10	90 ± 9	96 ± 8	72
Ethion	5	95	88 ± 8	93 ± 6	103 ± 7	87 ± 12	99 ± 8	105 ± 8	45
Fenpropathrin	5	110	83 ± 10	99 ± 7	110 ± 9	87 ± 11	102 ± 10	108 ± 10	22
Fenthion	1	79	79 ± 10	92 ± 7	105 ± 8	82 ± 11	102 ± 11	103 ± 10	35
Fenvalerate	1	87	79 ± 10	82 ± 8	100 ± 8	79 ± 15	88 ± 9	95 ± 10	42
HCH-a	1	88	73 ± 9	102 ± 7	110 ± 7	79 ± 12	98 ± 9	115 ± 9	59
HCH-b	1	95	78 ± 9	102 ± 6	109 ± 7	76 ± 14	99 ± 10	110 ± 10	62
Heptachlor	1	105	84 ± 11	94 ± 11	99 ± 8	85 ± 9	90 ± 11	104 ± 15	37
Hexachlorobenzene	1	119	86 ± 11	94 ± 10	93 ± 10	82 ± 15	87 ± 12	92 ± 12	70
Isophenphos methyl	5	89	86 ± 11	94 ± 10	93 ± 10	82 ± 15	87 ± 12	92 ± 12	27
Lindane (HCH gamma)	1	93	82 ± 12	94 ± 10	93 ± 10	82 ± 15	87 ± 12	92 ± 12	39
Paraoxon methyl	5	121	78 ± 11	96 ± 9	99 ± 9	82 ± 14	87 ± 11	95 ± 11	72
Phorate	5	114	73 ± 8	85 ± 7	94 ± 6	72 ± 11	88 ± 10	95 ± 10	68
Propyzamide	1	107	77 ± 9	85 ± 7	98 ± 7	80 ± 12	82 ± 8	99 ± 9	25
Quintozene	1	89	80 ± 8	89 ± 8	99 ± 6	78 ± 10	96 ± 10	100 ± 8	40
Tau-fluvalinate	1	102	84 ± 10	89 ± 7	92 ± 7	81 ± 12	88 ± 10	92 ± 9	58
Tetradifon	1	113	84 ± 10	82 ± 10	92 ± 9	81 ± 10	79 ± 12	88 ± 14	53
Tolclofos methyl	1	99	72 ± 10	78 ± 9	83 ± 8	70 ± 11	71 ± 10	81 ± 9	44
Trifluralin	5	91	79 ± 9	87 ± 4	93 ± 5	74 ± 12	87 ± 11	95 ± 10	38
Vinclozolin	1	108	84 ± 12	80 ± 6	78 ± 8	76 ± 14	83 ± 11	74 ± 12	97

*LOQs are presented in Table S1, with uncertainty of measurements (u′) at the LOQ not greater than 16% (expanded uncertainty U<32.5%). [a]: Recovery and Precision are reported for three different levels of the operative range (at LOQ, 10LOQ and 100LOQ)

Table S2. PFAS tentatively identified via *in silico* fragmentation using MetFrag Web tool in the honeybee samples

**No.**	**Monoisotopic Mass (Da)**	**Molecular Formula**	**MS/MS Fragment Ions (*m*/*z*)**
1	302.0790	$C_9H_{11}F_5N_4O_2$	150.031/285.041/286.049
2	338.1520	$C_{16}H_{22}F_4O_3$	233.081/295.132/309.148/133.064
3	282.0673	$C_{12}H_{11}F_5O_2$	221.039/263.050/219.023
4	250.0047	$C_{10}H_3F_5O_2$	221.002/178.991/206.987
5	272.1049	$C_{11}H_{16}F_4O_3$	152.010/124.015/238.063
6	301.1315	$C_{12}H_{19}F_4NO_3$	114.055/130.086/256.134
7	316.0948	$C_{10}H_{13}F_5N_4O_2$	180.005/239.035/285.041
8	324.0609	$C_{11}H_{11}F_7O_3$	171.044/211.076/255.066
9	330.0741	$C_{12}H_{14}F_4O6$	271.024/243.028/285.040
10	330.1104	$C_{14}H_{14}F_4N_4O$	180.065/119.049/197.060
11	351.0487	$C_{12}H_{11}F_7O_4$	211.039/268.038/283.061
12	353.0643	$C_{12}H_{13}F_7O_4$	285.076/252.042/138.031
13	357.0981	$C_{14}H_{18}F_4O_6$	253.050/271.061/103.039
14	365.1028	$C_{16}H_{18}F_4O_5$	253.050/197.060/107.013
15	395.0747	$C_{14}H_{15}F_7O_5$	224.047/253.047/285.076

Figure S1. TIC MRM chromatogram of a standard mix solution of pesticides at 50 ng/mL in LC-ESI-MS/MS

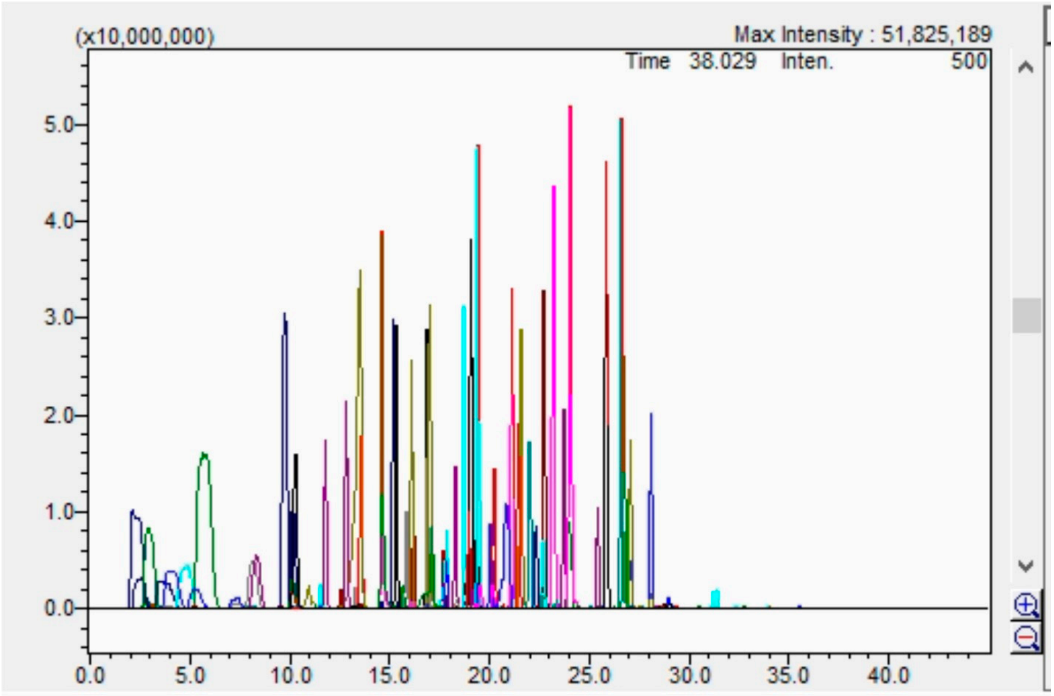

Figure S2. Triisopropanolamine annotation based on MS/MS fragmentation pattern using mzCloud database. The top panel is the MS/MS pattern of the honey bee extract, whereas the bottom panel is the MS/MS pattern of Triisopropanolamine from the mzCloud database.

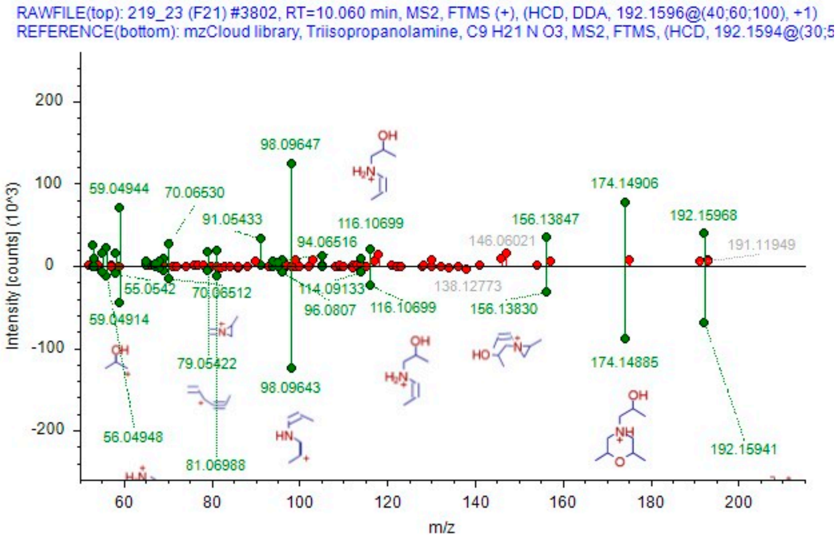

Figure S3. Acetophenone annotation based on MS/MS fragmentation pattern using mzCloud database. The top panel is the MS/MS pattern of the honey bee extract, whereas the bottom panel is the MS/MS pattern of Rimantadine from the mzCloud database.

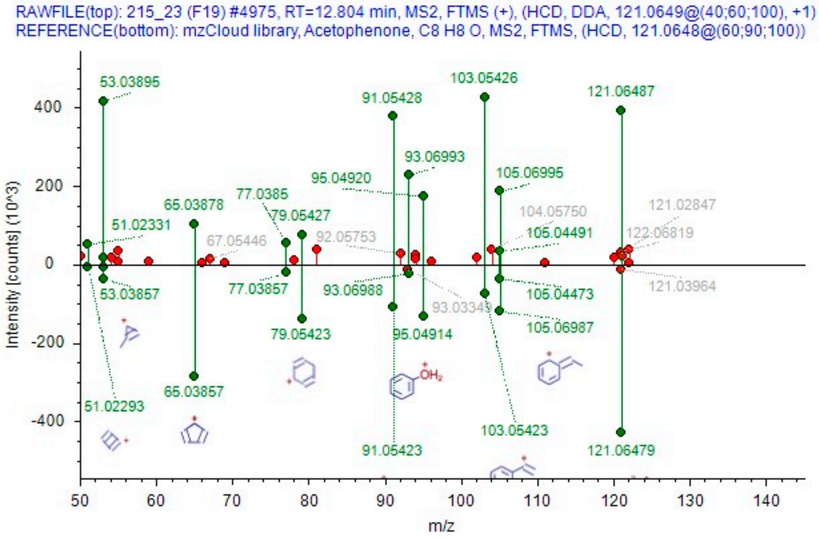

Figure S4. 1,5-Isoquinolinediol annotation based on MS/MS fragmentation pattern using mzCloud database. The top panel is the MS/MS pattern of the honey bee extract, whereas the bottom panel is the MS/MS pattern of 1,5-Isoquinolinediol from the mzCloud database.

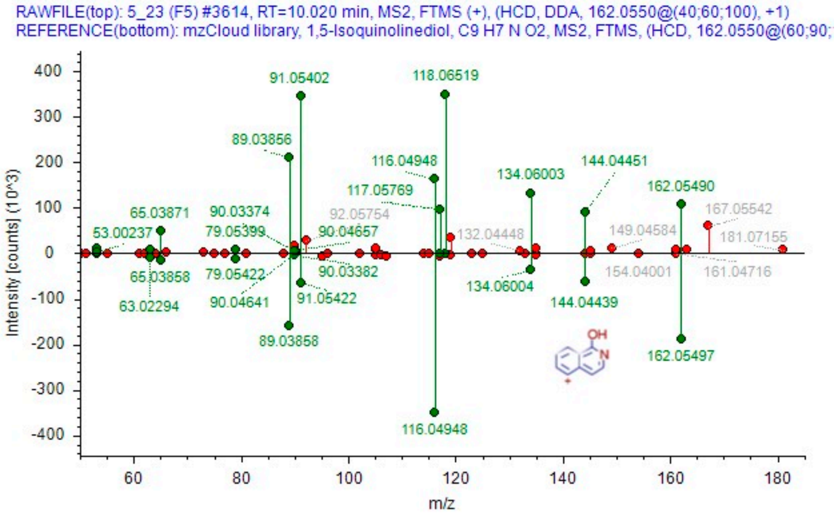

Figure S5. 1-Naphthol annotation based on MS/MS fragmentation pattern using mzCloud database. The top panel is the MS/MS pattern of the honey bee extract, whereas the bottom panel is the MS/MS pattern of 1-Naphthol from the mzCloud database.

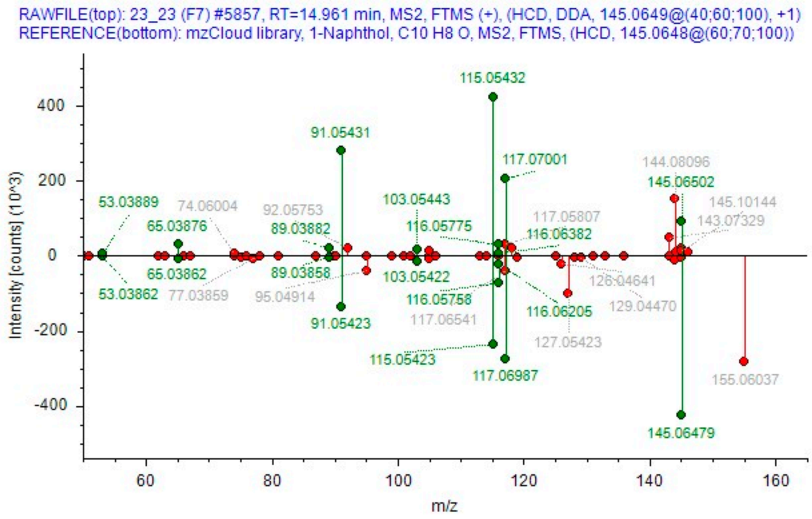

Figure S6. 2,2-Methylenebis(4-ethyl-6-tert-butylphenol) annotation based on MS/MS fragmentation pattern using mzCloud database. The top panel is the MS/MS pattern of the honey bee extract, whereas the bottom panel is the MS/MS pattern of 2,2-Methylenebis(4-ethyl-6-tert-butylphenol) from the mzCloud database.

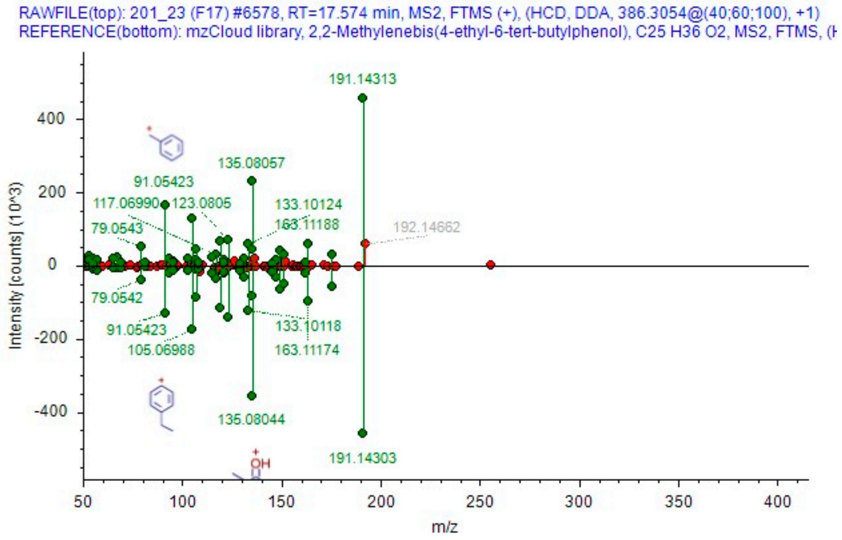

https://doi.org/10.3390/xxxxx

Figure S7. 2-Amino-6-methylmercaptopurine annotation based on MS/MS fragmentation pattern using mzCloud database. The top panel is the MS/MS pattern of the honey bee extract, whereas the bottom panel is the MS/MS pattern of 2-Amino-6-methylmercaptopurine from the mzCloud database.

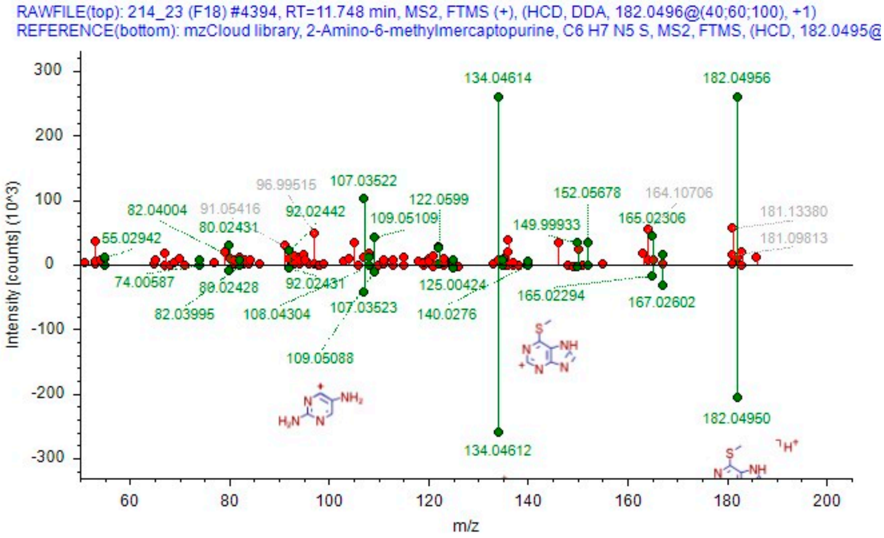

Figure S8. Arachidonic acid annotation based on MS/MS fragmentation pattern using mzCloud database. The top panel is the MS/MS pattern of the honey bee extract, whereas the bottom panel is the MS/MS pattern of Arachidonic acid from the mzCloud database.

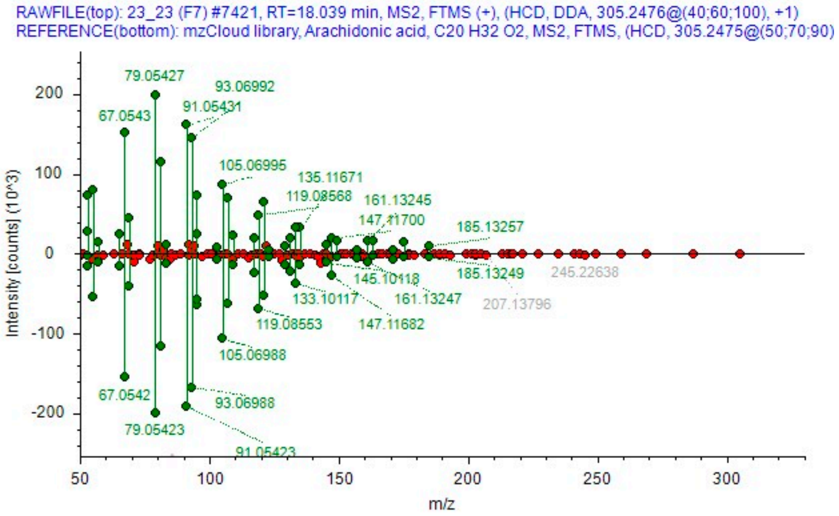

https://doi.org/10.3390/xxxxx

Figure S9. Betulinic acid methyl ester annotation based on MS/MS fragmentation pattern using mzCloud database. The top panel is the MS/MS pattern of the honey bee extract, whereas the bottom panel is the MS/MS pattern of Betulinic acid methyl ester from the NIST database.

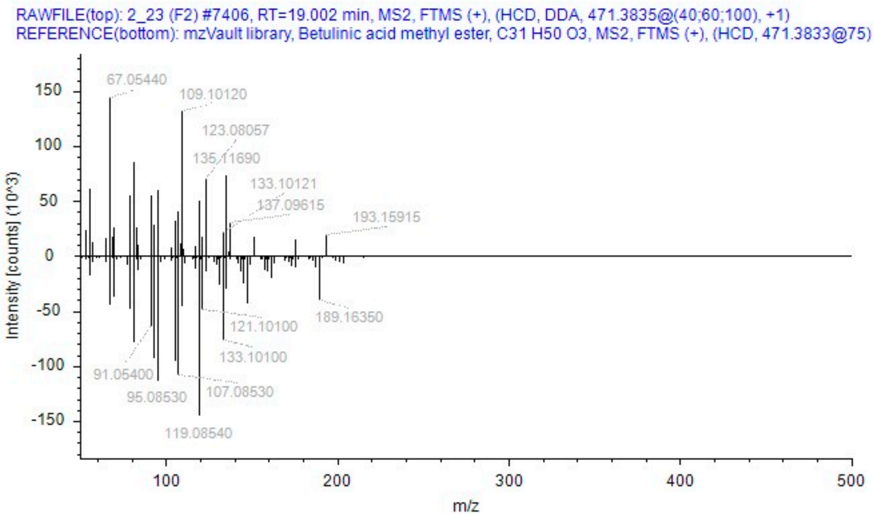

Figure S10. Deoxylimonin annotation based on MS/MS fragmentation pattern using mzCloud database. The top panel is the MS/MS pattern of the honey bee extract, whereas the bottom panel is the MS/MS pattern of Deoxylimonin from the mzCloud database.

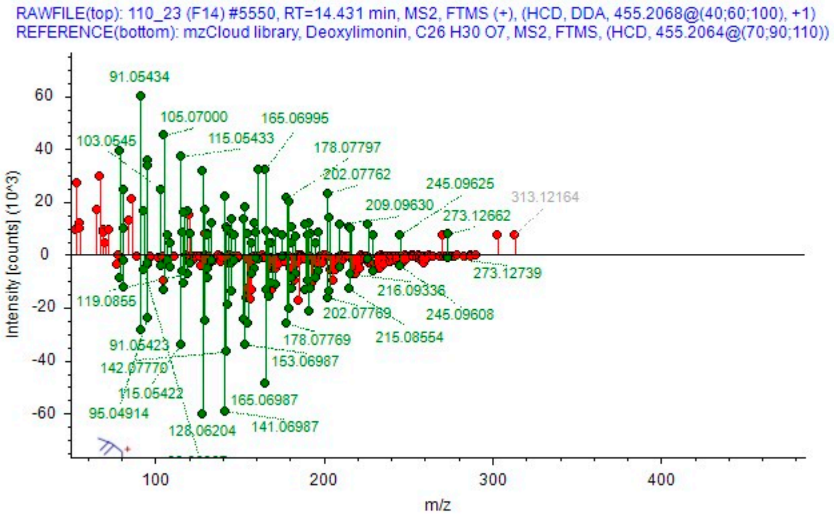

Figure S11. Diisobutylphthalate annotation based on MS/MS fragmentation pattern using mzCloud database. The top panel is the MS/MS pattern of the honey bee extract, whereas the bottom panel is the MS/MS pattern of Diisobutylphthalate from the mzCloud database.

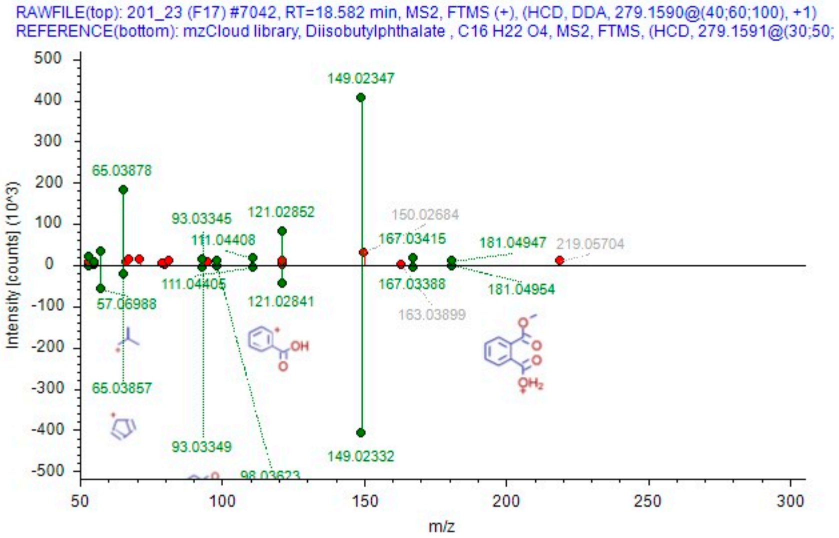

Figure S12. Dimethyl sebacate annotation based on MS/MS fragmentation pattern using mzCloud database. The top panel is the MS/MS pattern of the honey bee extract, whereas the bottom panel is the MS/MS pattern of Dimethyl sebacate from the mzCloud database.

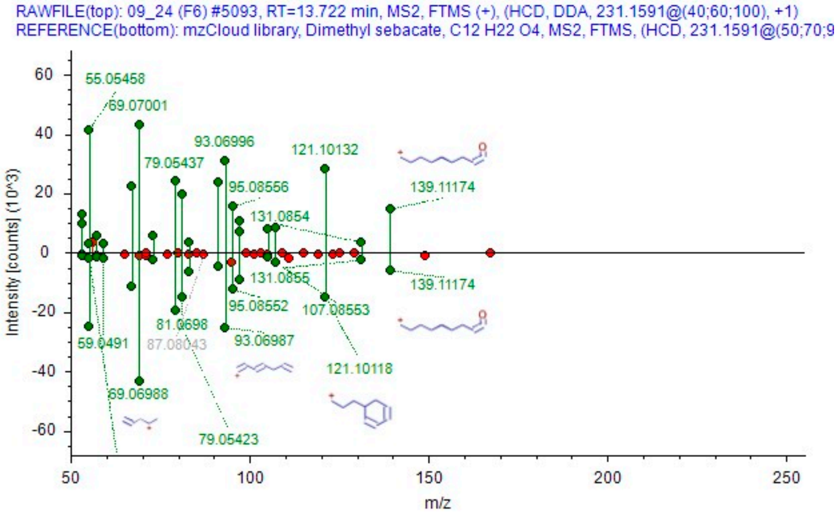

Figure S13. D-Panthenol annotation based on MS/MS fragmentation pattern using mzCloud database. The top panel is the MS/MS pattern of the honey bee extract, whereas the bottom panel is the MS/MS pattern of D-Panthenol from the mzCloud database.

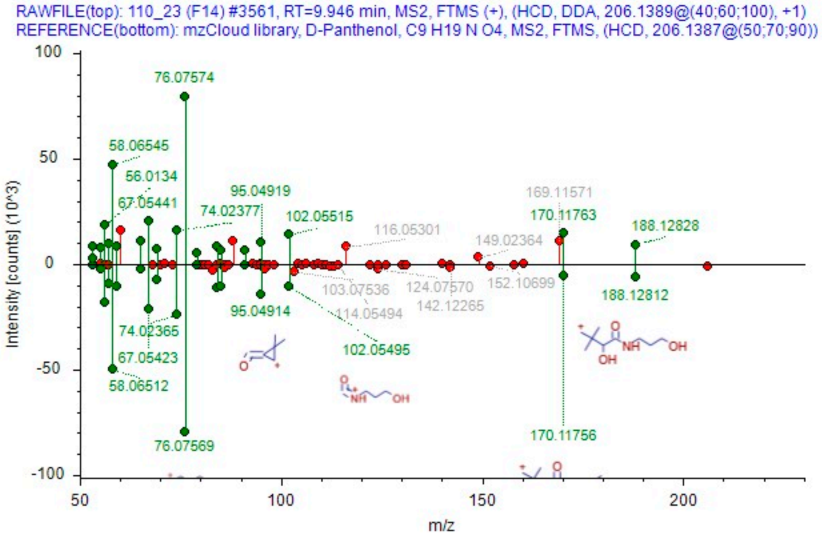

Figure S14. Ethyl oleate annotation based on MS/MS fragmentation pattern using mzCloud database. The top panel is the MS/MS pattern of the honey bee extract, whereas the bottom panel is the MS/MS pattern of Ethyl oleate from the mzCloud database.

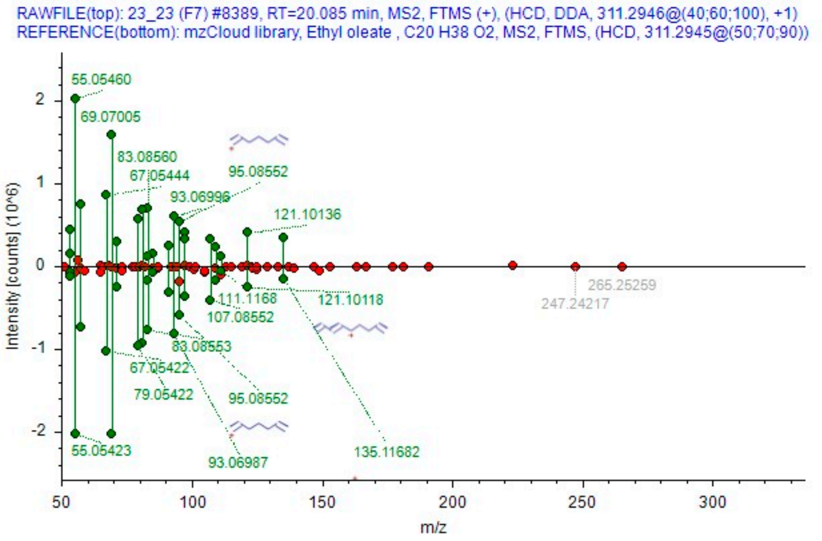

Figure S15. Fenbendazole annotation based on MS/MS fragmentation pattern using mzCloud database. The top panel is the MS/MS pattern of the honey bee extract, whereas the bottom panel is the MS/MS pattern of Fenbendazole from the mzCloud database.

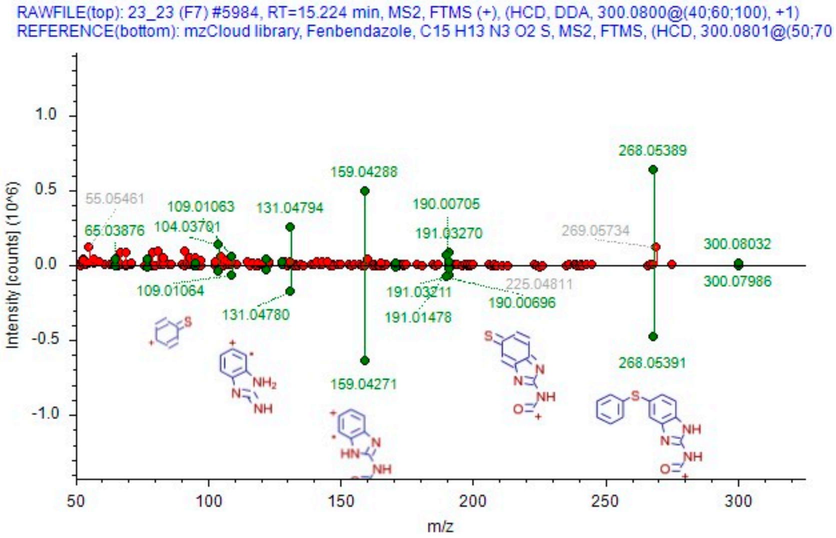

Figure S16. Gabapentin annotation based on MS/MS fragmentation pattern using mzCloud database. The top panel is the MS/MS pattern of the honey bee extract, whereas the bottom panel is the MS/MS pattern of Gabapentin from the mzCloud database.

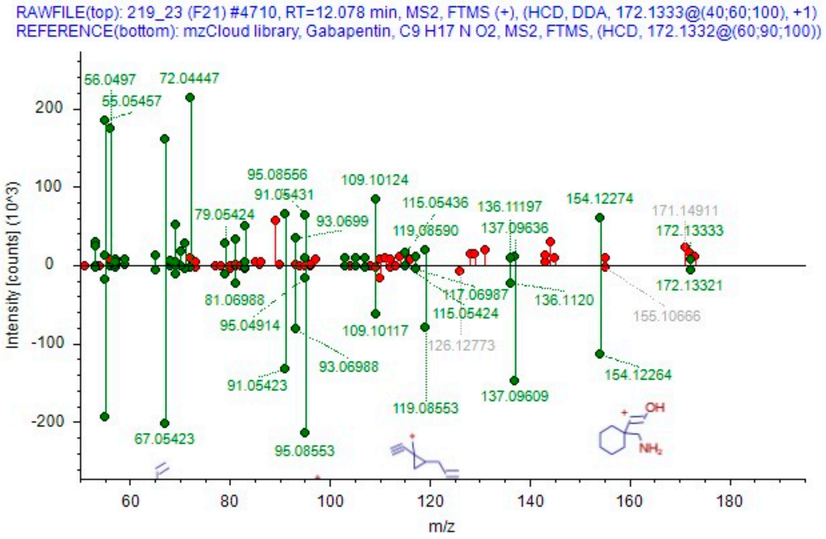

Figure S17. Icaridin annotation based on MS/MS fragmentation pattern using mzCloud database. The top panel is the MS/MS pattern of the honey bee extract, whereas the bottom panel is the MS/MS pattern of Icaridin from the mzCloud database.

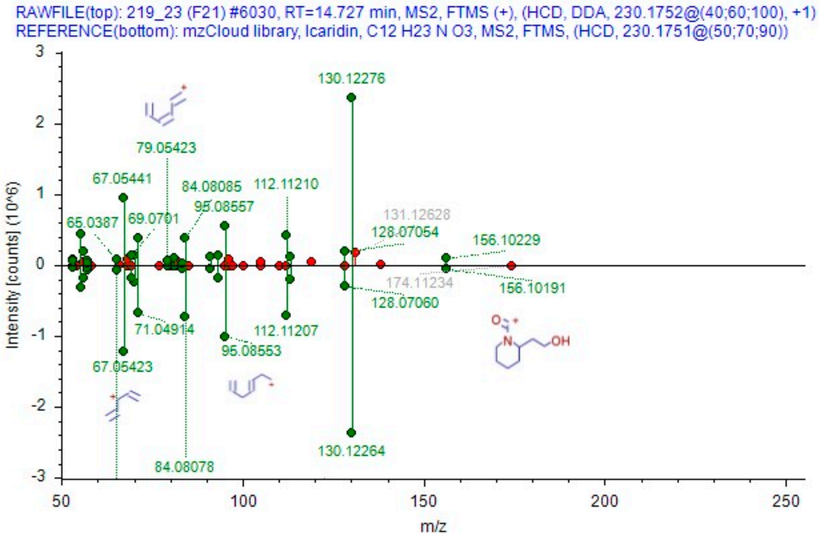

Figure S18. Isobornyl methacrylate annotation based on MS/MS fragmentation pattern using mzCloud database. The top panel is the MS/MS pattern of the honey bee extract, whereas the bottom panel is the MS/MS pattern of Isobornyl methacrylate from the mzCloud database.

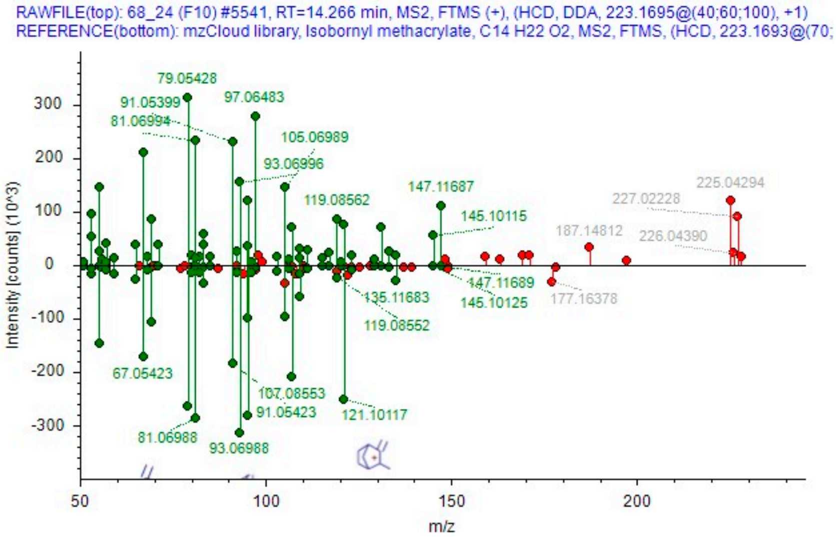

Figure S19. Limonin annotation based on MS/MS fragmentation pattern using mzCloud database. The top panel is the MS/MS pattern of the honey bee extract, whereas the bottom panel is the MS/MS pattern of Limonin from the mzCloud database.

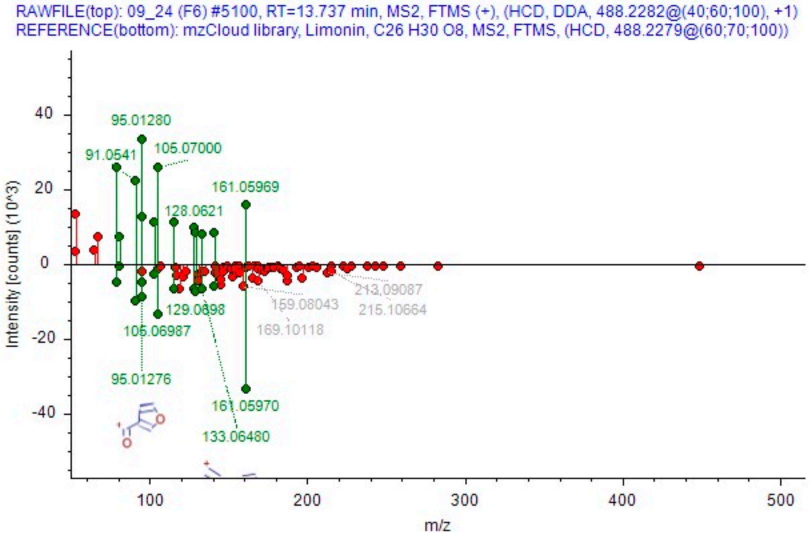

Figure S20. N-Butylbenzenesulfonamide annotation based on MS/MS fragmentation pattern using mzCloud database. The top panel is the MS/MS pattern of the honey bee extract, whereas the bottom panel is the MS/MS pattern of N-Butylbenzenesulfonamide from the mzCloud database.

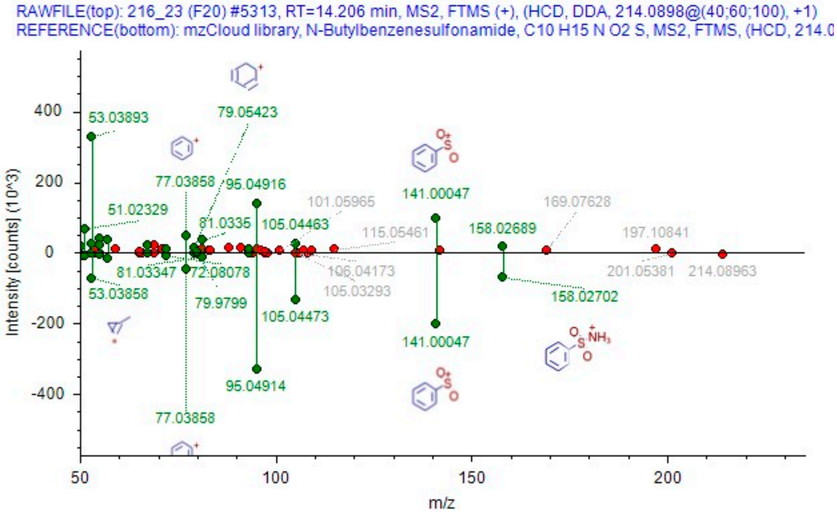

Figure S21. Noscapine annotation based on MS/MS fragmentation pattern using mzCloud database. The top panel is the MS/MS pattern of the honey bee extract, whereas the bottom panel is the MS/MS pattern of Noscapine from the mzCloud database.

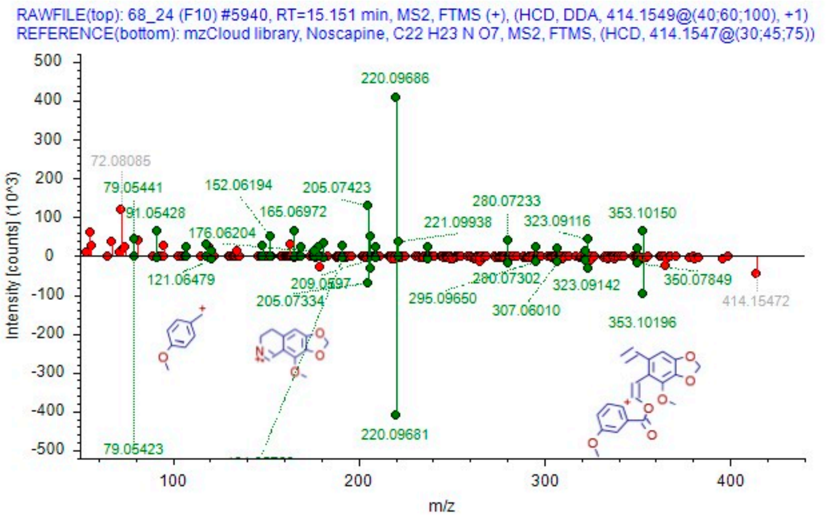

Figure S22. Scopolamine annotation based on MS/MS fragmentation pattern using mzCloud database. The top panel is the MS/MS pattern of the honey bee extract, whereas the bottom panel is the MS/MS pattern of Scopolamine from the mzCloud database.

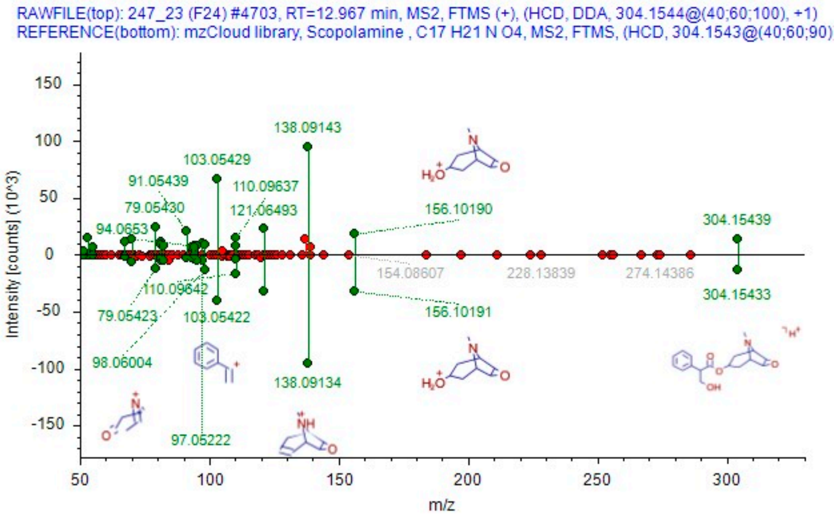

Figure S23. Skatole annotation based on MS/MS fragmentation pattern using mzCloud database. The top panel is the MS/MS pattern of the honey bee extract, whereas the bottom panel is the MS/MS pattern of Skatole from the mzCloud database.

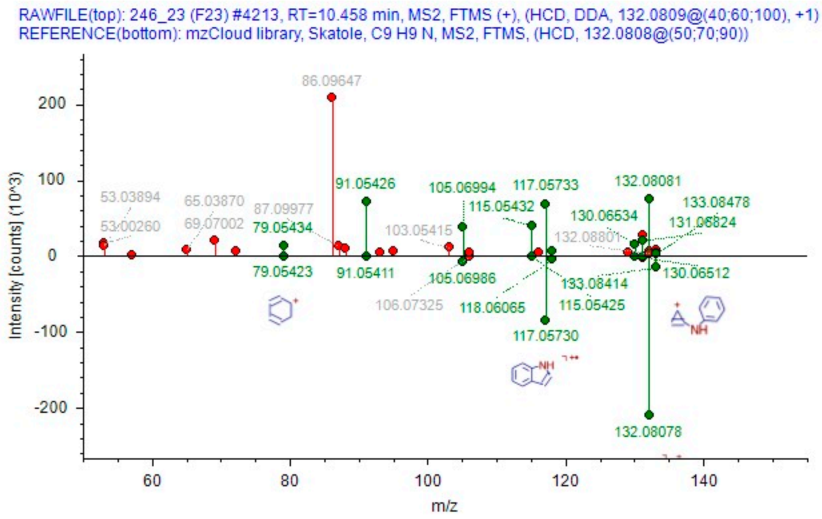

Figure S24. Stylopine annotation based on MS/MS fragmentation pattern using mzCloud database. The top panel is the MS/MS pattern of the honey bee extract, whereas the bottom panel is the MS/MS pattern of Stylopine from the NIST database.

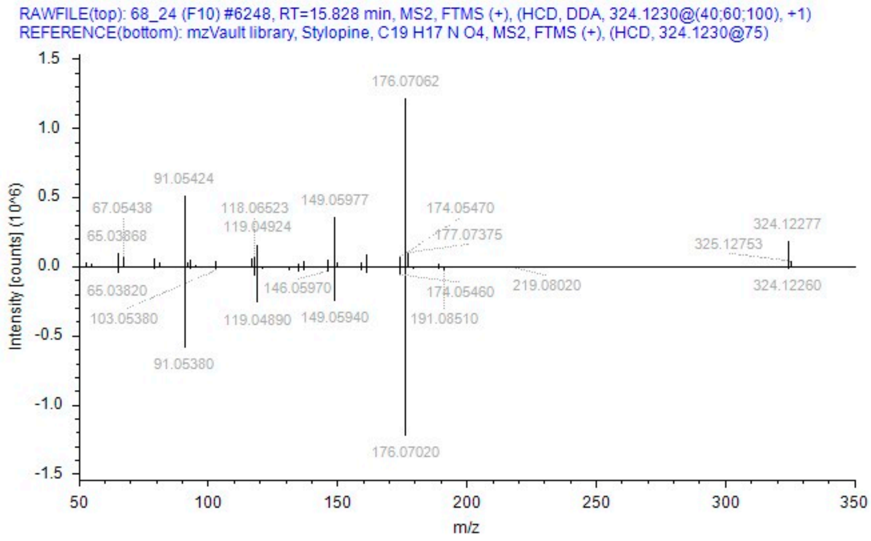

Figure S25. Syringate annotation based on MS/MS fragmentation pattern using mzCloud database. The top panel is the MS/MS pattern of the honey bee extract, whereas the bottom panel is the MS/MS pattern of Syringate from the NIST database.

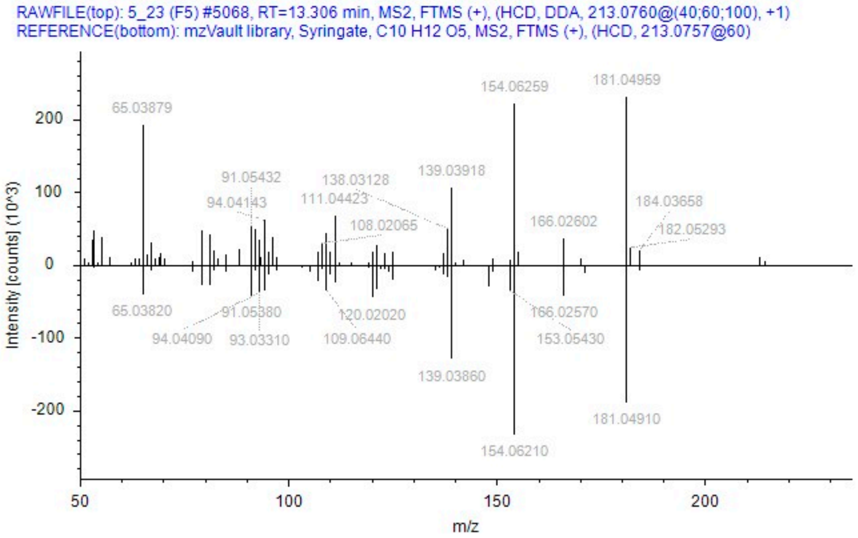

Figure S26. Trans-Cinnamaldehyde annotation based on MS/MS fragmentation pattern using mzCloud database. The top panel is the MS/MS pattern of the honey bee extract, whereas the bottom panel is the MS/MS pattern of trans-Cinnamaldehyde from the mzCloud database.

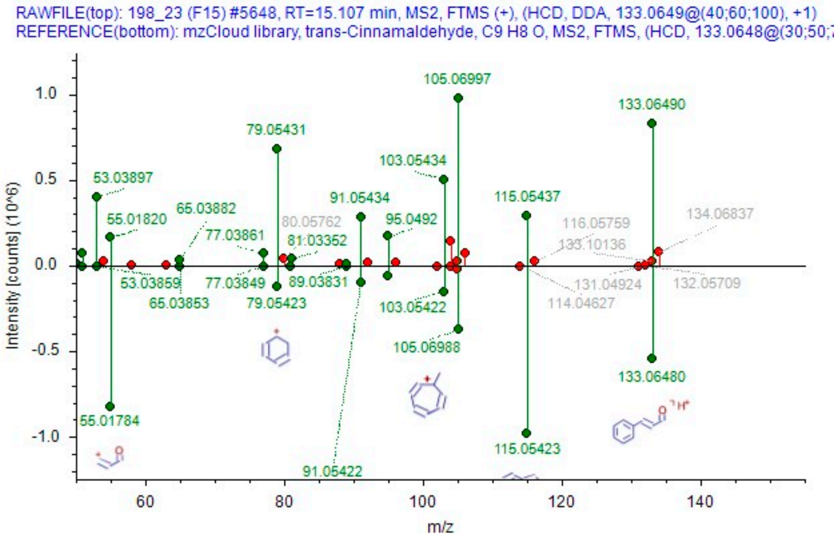

Figure S27. MRM chromatograms of phenothrin detection in bee sample (108-23)

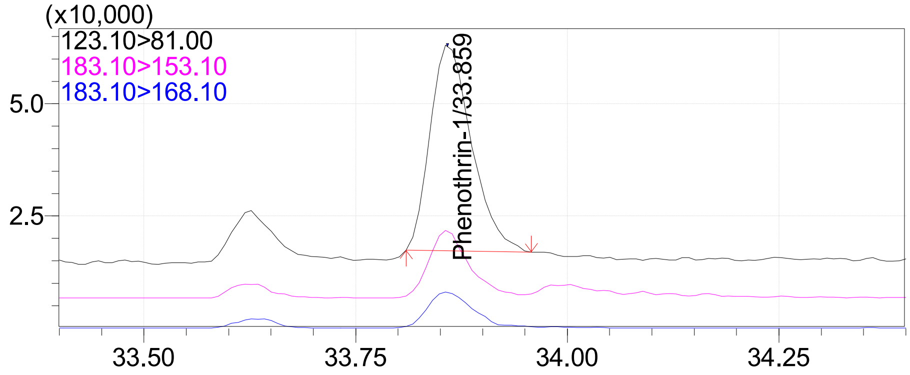

Figure S28. Potential chemical structures for PFAS compounds 2 and 14

Compound 2

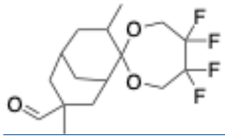

Compound 14

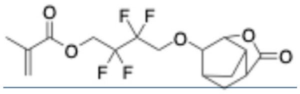

References

Matuszewski, B. K., et al., 2003. Strategies for the assessment of matrix effect in quantitative bioanalytical methods based on HPLC-MS/MS. Analytical Chemistry. 75**,** 3019-3030.
